# Climate sustainability through a dynamic duo: Green hydrogen and crypto driving energy transition and decarbonization

**DOI:** 10.1073/pnas.2313911121

**Published:** 2024-03-25

**Authors:** Apoorv Lal, Fengqi You

**Affiliations:** ^a^Systems Engineering, College of Engineering, Cornell University, Ithaca, NY 14853; ^b^Robert Frederick Smith School of Chemical and Biomolecular Engineering, Cornell University, Ithaca, NY 14853

**Keywords:** climate change, green hydrogen, renewable energy, blockchain

## Abstract

Climate change persists as a pressing global issue due to high greenhouse gas emissions from fossil fuel–based energy sources. A transition to a greener energy matrix combined with carbon offsetting is imperative to mitigate the rate at which global temperature ascends. While countries have deployed faith in green hydrogen to accelerate worldwide decarbonization efforts, the concurrent rise of blockchain-operated crypto-applications, such as bitcoin, has exacerbated climate change concerns. In this study, we propose technological solutions that combine the green hydrogen infrastructure with bitcoin mining operations to catalyze environmental and socioeconomic sustainability in climate change mitigation strategies. Since the present state of crypto-operations undeniably contributes to worldwide carbon emissions, it becomes vital to explore opportunities for harnessing the widespread enthusiasm for bitcoin as an aid toward a sustainable and climate-friendly future. Our findings reveal that green hydrogen production, paired with crypto-operations, can accelerate the deployment of solar and wind power capacities to boost conventional mitigation frameworks. Specifically, leveraging the economic potential derived from green hydrogen and bitcoin for incremental investment in renewable energy penetration, this dynamic duo can enable capacity expansions of up to 25.5% and 73.2% for solar and wind power installations. Therefore, the proposed technological solutions that leverage green hydrogen and bitcoin mining, bolstered with appropriate policy interventions, can not only strengthen renewable power generation and carbon offsetting capacities but also contribute significantly to achieving climate sustainability.

## Significance

This work introduces a strategy by synergizing green hydrogen technologies with bitcoin mining operations to address the concurrent challenges of cryptocurrency growth and climate change. We highlight the potential of this dynamic duo to enhance the deployment of renewable energy sources, particularly solar and wind power. By proposing a framework incorporating blockchain-driven crypto-applications with sustainable energy solutions, this work underlines the transformative potential of emerging technologies in the global pursuit of climate sustainability. Supported by appropriate policy measures, this integrated systems approach can augment renewable power generation and carbon offsetting capacities, advancing toward a sustainable and climate-resilient future.

Climate change patterns are primarily attributed to increased greenhouse gas (GHG) emissions due to anthropogenic activities and natural systems. Energy-derived carbon dioxide (CO_2_) emissions continue to drive the global carbon debt (1, 2), with fossil fuels persisting as the primary energy source (3–6). This concerning trend underscores the urgency of reducing carbon emissions across various sectors, which has led to the development of several pathways for climate change mitigation (7–9). To mitigate fossil-based GHG emissions, countries must deploy renewable energy sources (10–12), a crucial component of the conventional mitigation framework that aids in accelerating the energy transition (7). Even with the implementation of targeted policies, incentives, and risk reduction measures (13), this transition to a cleaner energy structure faces obstacles such as high capital investment and the intermittent nature of renewable energy sources (14–16). As an extension of the conventional mitigation framework, several countries also plan to use an energy carrier to import clean energy (17–19). Green hydrogen, in particular, is expected to play a pivotal role in mitigating climate change by supplying renewable energy (20–22). It has received extensive policy support for the deployment of electrolyzer infrastructure and self-sufficient renewable capacity (23–26), and the rising demand for carbon-free hydrogen is expected to boost international hydrogen trade (27–31). However, the production of traditional carriers results in energy inefficiencies and losses along with direct carbon emissions based on the fuel used in transportation (32–34). Despite the inherent limitations of traditional energy carriers, green hydrogen production can support renewable power facilities while catering to the growing hydrogen demand derived from clean energy sources.

While transitioning from a fossil-driven economy to a hydrogen one is expected to secure substantial regulatory endorsement in decarbonization initiatives, the past decade has also witnessed a significant increase in energy expenses of blockchain-based applications. Historically, fossil-powered blockchain applications have exacerbated the climate change problem, with some cryptocurrencies, such as bitcoin, having an energy demand comparable to Argentina (35). The dominance of grid-powered mining in the crypto industry has resulted in staggering carbon debt, which continues to grow at a steady rate (36–39). By shifting the power source of cryptocurrencies to promote renewable infrastructure deployment, an essential link within the clean energy value chain can be established. Correspondingly, negative mitigation technologies offer a promising way to decarbonize various sectors, particularly through carbon offsetting, complementing the conventional climate mitigation framework (40, 41). Direct air capture (DAC), a cost-intensive technology, can be utilized to attain carbon neutrality for cryptocurrency mining (42). Moreover, legislative actions, such as the inflation reduction act (IRA) (43–45), can facilitate a clean power supply to mining operations and reduce dependence on the grid. By implementing these measures, cryptocurrency mining can potentially be operated with net-zero GHG emissions, and the resulting economic potential can be harnessed to facilitate climate change mitigation.

In this work, we hypothesize that the dynamic duo comprising green hydrogen and bitcoin can accelerate the widespread deployment of renewable energy sources, facilitate the implementation of carbon offsetting mechanisms, and incentivize sustainable practices in the energy sector. To test the hypothesis, this study aims to investigate a systematic multipronged strategy for conventional climate change mitigation in US states, utilizing renewable power facilities to mine bitcoin and produce green hydrogen. Harnessing renewable energy for mining activities and green hydrogen production can yield dual benefits, both economic and environmental. This approach can minimize emissions tied to the consumption of fossil fuels and expedite the widespread adoption of renewable energy sources. Additionally, we propose a technological solution of using crypto-operations as virtual energy carriers centered on the development of renewable infrastructure to supply clean energy across diverse locations. Virtual energy carriers offer an efficient and interchangeable alternative to traditional energy carriers to import clean energy, avoiding energy losses from equipment inefficiencies and eliminating transportation-related carbon emissions. This study presents a comparative analysis of renewable power utilization, considering a traditional energy carrier like green hydrogen and bitcoin as a virtual carrier. Last, we examine scenarios for grid-powered crypto-operations with green hydrogen power supply across different US states under carbon-neutral conditions for augmenting the negative mitigation framework. Specifically, the scenarios presented here analyze the economic potential of this combined operation, which can enhance the carbon offsetting capacity and serve as a catalyst for decarbonization endeavors.

The conventional mitigation framework relies on enhanced renewable energy penetration to mitigate fossil-based GHG emissions. This work investigates how the profitability from the combined operation of bitcoin mining and green hydrogen production powered through renewable energy sources can increase capital investment in the conventional mitigation framework. We consider the development of solar and wind power facilities across various US states, which subsequently provide power to mining and auxiliary equipment, as well as the green hydrogen infrastructure. The proposed technological solution seeks to capitalize on the growing demand for carbon-free hydrogen and cryptocurrencies to enhance profitability. This increased profitability can then be channeled into capital investment for renewable energy infrastructure, ultimately bolstering the conventional mitigation framework. In addition, we consider future scenarios to assess the effectiveness of our approach in light of anticipated advancements in renewable energy technology. Overall, the study's methodology presents a comprehensive strategy that synergistically links both operations to increase profits, which can then be utilized as a capital investment in renewable energy infrastructure, thereby strengthening the conventional mitigation framework.

As an extension of the conventional mitigation framework, we examine the potential of utilizing crypto-operations, such as bitcoin, as virtual energy carriers that can leverage their monetary value for renewable power generation in diverse settings. We initiate our investigation with a comparative analysis of the percentage utilization of available power, focusing on renewable power installations to produce a traditional energy carrier like green hydrogen. This work also finds the percentage utilization achievable if crypto-operations like bitcoin were integrated with solar and wind facilities with identical specifications. Subsequently, we employ the levelized cost of power generation to evaluate the amount of clean energy that can be funded through the monetary value of cryptocurrencies as virtual carriers. Furthermore, we explore the effectiveness of the proposed technological solution of a virtual carrier in future scenarios, utilizing projections of technological advancements that reduce levelized power generation costs. This technological solution demonstrates that using crypto-operations as virtual energy carriers presents a promising strategy for enhancing the utilization of clean energy sources and fostering the growth of renewable-powered infrastructure.

The adoption of negative mitigation technologies holds the potential for advancing decarbonization efforts in multiple sectors by implementing carbon offsetting that effectively supports the conventional mitigation framework. We assess the possibility of using traditional grid-power supply for miners, combined with green hydrogen power generation, to mine carbon-neutral bitcoins. The monetary value of the mined currency can then be used to fund the capture of CO_2_ based on the levelized cost of carbon capture technologies, ultimately advancing the negative mitigation framework. This work takes into account the bitcoin mining setup in different states, consisting of miners and auxiliary equipment operated on the grid power supply, as well as the impact of IRA on the green hydrogen power input. We consider a base case evaluation with no incentives for green hydrogen power input and an incentivized scenario to evaluate the profitability of carbon-neutral mining and the investment in negative mitigation technologies. The incentivized scenario examines the role of tax credits in enhancing the profitability of carbon-neutral mining and subsequent investment in negative mitigation technologies. This study also considers how different degrees of incentivization can lower the costs of green hydrogen power generation and, consequently, the total carbon capture funded by the profits generated from bitcoins.

The key findings of this work include the following:

• **Propel decarbonization of the grid:** Energy transition can be empowered through the economic potential of the proposed multipronged strategy, which enables a one-megawatt initial investment in rated capacity into 1.25 MW of solar and 1.73 MW of wind energy capacity.
• **Accelerated “Climate-Greening” Potential:** Technological advancements allow this dynamic duo of green hydrogen and bitcoin to enhance the effectiveness of the multipronged strategy for conventional climate mitigation framework by 149% in solar power capacity and a 140% increase in wind power capacity by 2050.
• **Additional generation capacity:** Leveraging crypto as virtual carriers can enhance renewable capacity and potentially achieve up to 98% and 92% utilization of available solar and wind power, respectively.
• **Climate Laws:** As a boost to the decarbonization efforts in various sectors, the IRA can incentivize US states to generate green hydrogen power and mine carbon-neutral bitcoins, which could promote a minimum negative mitigation capacity of 7.4 tCO_2_-eq per bitcoin mined.
• **State-specific “climate incentivization”:** A substantial proportion of renewable energy sources in the power grid and favorable electricity prices enable states such as Idaho to support 22.6 tCO_2_-eq carbon capture capacity for every bitcoin mined.

The policy implications of this work can be summarized as follows:

• **Climate goals:** The convergence of technological advancements in renewable energy and green hydrogen infrastructure, combined with cryptocurrency value, can accelerate renewable energy penetration to create a more sustainable energy landscape.
• **Energy policy:** By utilizing crypto-operations as virtual energy carriers, policymakers can significantly increase renewable energy capacity in diverse settings while minimizing energy losses from equipment inefficiencies and eliminating transportation-related carbon emissions.
• **Environmental policy:** Providing policy support to green hydrogen power generation and supporting states with low fossil fuel dependence can simultaneously decarbonize crypto-operations and support the negative mitigation framework.

## Materials and Methods

### Data Collection and Sources.

The availability of appropriate data sources is crucial to examine the feasibility of the proposed technological solutions. The System Advisor Model by the National renewable energy laboratory (NREL) and the Visual Crossing Weather Application Programming Interface have been used to get the wind speeds and solar irradiation intensities to calculate power generation in different states (46, 47). The projected values for the cost parameters, such as levelized cost, capital expenditure, and operational and maintenance costs for solar and wind power generation facilities, were obtained from NREL’s Annual Technology Baseline documentation (48). The other important data required, including the bitcoin prices and the network difficulties, have been obtained from the available mining database (49). The mining database was also used to get the network specifications, including the geographical distribution of mining computational power (50). Correspondingly, specifications for the mining equipment match the market data for the available miners (51). Regarding estimating the avoided emissions based on the analysis of life cycle emissions, we utilize the ecoinvent database to get the characterization factors (52). The contribution of the different hydrogen pathways in meeting the global demand is taken from the previous works for the avoided emission calculations (53, 54). The other essential parameters for the study, including the equipment specifications, have also been obtained from previous literature (42, 55–64).

### Optimization Modeling Framework.

In this section, we introduce the optimization modeling framework used to assess the different parts of the study. The general systems optimization framework used in the study for the proposed technological solutions is presented below.

**Table:** 

max	NPV of proposed technological solutions
s.t.	Load balance constraints given in *SI Appendix*, Eqs. **S1**–**S5**, **S33**–**S35**, **S53**–**S54**, and **S70**–**S71**
	Operational constraints given in *SI Appendix*, Eqs. **S6**–**S16**, **S36**–**S39**, **S55**–**S59**, and **S72**–**S75**
	Economic evaluation constraints given in *SI Appendix*, Eqs. **S17**–**S32**, **S40**–**S52**, **S60**–**S69**, and **S76**–**S82**

The load balance constraints utilize the data for wind speed and solar irradiation for the different states installing a renewable power generation facility with a fixed capacity. The calculated wind or solar power values indicate the total available power distributed among the utilized and surplus power at different time intervals. Similar load balance constraints are applicable in the comparative analysis of traditional and virtual carrier operations. However, these constraints vary in the negative mitigation framework. The total available power represents the power imported from the respective state electricity grids and power generation using green hydrogen. The operational constraints in the optimization framework govern the equipment performance. For example, the operation of mining equipment leads to the generation of heat, which must be removed using heat pumps. The power consumption in the heat pumps can be estimated using the specified coefficient of performance. These constraints include the energy supply from traditional carriers and the CO_2_ capture quantities from DAC units, which are calculated based on equipment efficiencies. Correspondingly, these also specify the maximum limit on the power dedicated to different equipment based on the number of units utilized and the individual capacities. The economic evaluation in the study estimates the total revenue for the project based on the summation of the income for different time intervals, including the revenue generated for mining bitcoin and green hydrogen production for the respective scenarios. The income generated from the crypto mining process depends on the price of the currency, the number of coins rewarded on adding a new block, power dedicated to mining equipment, and the current network difficulty. The capital expenditure for the different process components is computed using unit capital cost and the number of units utilized. The operating cost for the process components is estimated by summing the operational and maintenance cost units in different time intervals or as the percentage of the capital cost for each year of operation. Some components in the total operating cost are the storage and transportation cost for the captured CO_2_ or the transportation cost of the produced carrier. Based on the considered project life, we utilize the double-depreciation method to calculate the corresponding salvage values for the equipment used.

In the current practice, the bitcoin industry mainly uses the fossil-dependent grid power supply to meet energy expenses, leading to staggeringly high carbon emissions. Also, global hydrogen production is dominated by processes such as steam methane reforming and coal gasification. Accordingly, if a given solar or wind power facility mines bitcoin or produces green hydrogen, this shift from traditional methods to renewable sources corresponds to a substantial amount of avoided emissions that would have been released during usual operations. In order to calculate the total avoided emissions corresponding to the implementation of technological solutions, we utilize the emissions factors for the different process sections. For instance, in the case of conventional and negative mitigation frameworks incorporating crypto-operations and green hydrogen production, the percentage contribution of traditional fossil-based hydrogen production pathways and their respective emission factors can be used to calculate the total emissions avoided due to green hydrogen production. Correspondingly, using the hash rate distribution for the given cryptocurrency, the emission factors for the grid power supply, and the percentage of miners in that location, the emissions associated with mining the same number of coins can be calculated. We leverage the significant economic potential from crypto-operations in the proposed technological solutions as the driving factor to enhance climate mitigation efforts. For instance, maximum economic benefits derived from the conventional mitigation framework can be used to enable the highest increment in solar and wind power capacity installations. Similarly, the effectiveness of the negative mitigation framework relies on the total carbon capture capacity, which can be attained using the economic potential generated. Therefore, the single-objective optimization modeling framework maximizes the economic potential, underscoring economics as the driving force for achieving maximum deployment in climate mitigation frameworks through technological solutions considered in the study.

### Life Cycle Assessment (LCA).

In this work, the LCA methodology is employed to assess the environmental impacts of optimal solutions derived from the conventional and negative mitigation frameworks and the application of traditional and virtual carriers for climate change mitigation. This approach evaluates the detailed environmental implications associated with achieving the maximum capacity expansion in the climate mitigation frameworks, leveraging the economic potential generated from the technological solutions as the driving force. The analysis within the conventional mitigation framework evaluates the environmental impacts associated with solar and wind-powered bitcoin mining and green hydrogen production. Due to the multifunctionality inherent in the framework, where bitcoin mining and green hydrogen infrastructure operate on renewable power supply, the environmental impacts are evaluated based on energy allocation. This dual functionality necessitates the allocation based on renewable energy supply to ensure that the distinct environmental contributions of bitcoin mining and hydrogen production are reflected. Similar to the conventional mitigation framework, this work investigates the environmental impacts associated with bitcoin as a virtual energy carrier based on the renewable energy sources harnessed in cryptocurrency operations. Furthermore, this framework contrasts with traditional energy carriers, where the role of transportation is also included. In this case, the LCA is adapted to evaluate impacts based on energy that can be supplied by the traditional energy carrier. Last, in the case of the negative mitigation framework, the environmental impacts are analyzed based on the percentage utilization of grid power supply and green hydrogen–based power generation, considering the diverse electricity mix of different US states. The comprehensive assessment of the negative mitigation framework highlights the distribution between utilizing grid infrastructure and green hydrogen technologies within state-specific contexts to influence the environmental impacts of each bitcoin mined.

**Fig. 1.**
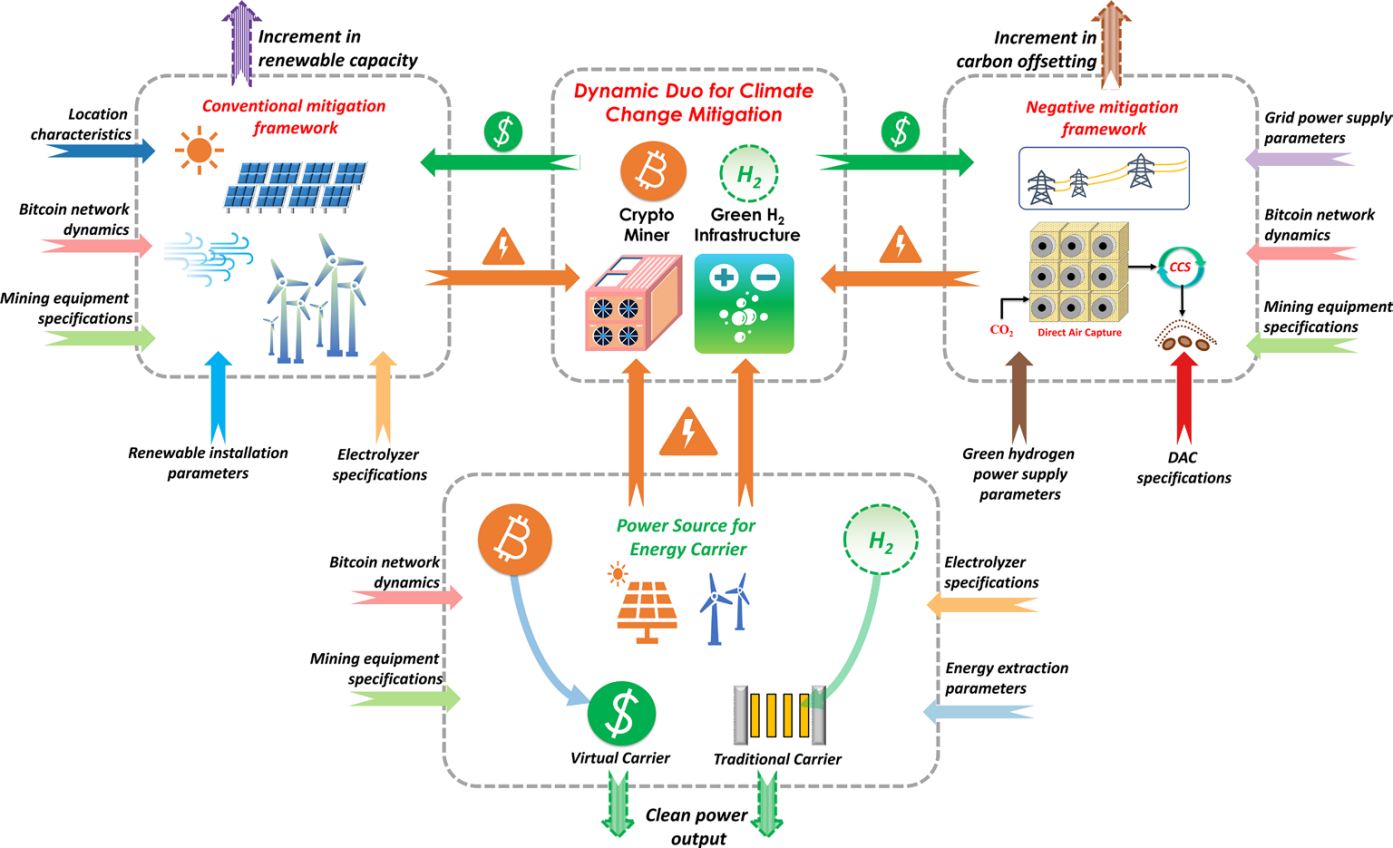
Systems analysis framework for examining the potential of green hydrogen and bitcoin as a dynamic duo to strengthen both conventional and negative mitigation strategies while serving as traditional and virtual carriers for energy conversion, respectively.

The Life Cycle Inventory (LCI) was tailored to evaluate the environmental impacts associated with the various climate mitigation strategies in this work. For the conventional mitigation framework and the assessment of virtual and traditional carriers, LCI data included the power generation using renewable energy sources, incorporating specific performance metrics of wind turbines and solar PV, which were crucial in calculating the environmental impacts of renewable energy generation. Among the green hydrogen technologies, the operating parameters for the Alkaline Water Electrolyzer were used since it has among the highest technological maturity and commercial outreach (65–67). In the case of DAC technologies, we utilize the solid-sorbent DAC since it can operate solely on electricity derived from clean energy sources (68) and requires low regeneration temperatures (69). The equipment specifications corresponding to the green hydrogen infrastructure, DAC, and mining operations used as part of the LCI have been depicted in *SI Appendix*, Table S3, along with sensitivity analysis results to investigate the role of alternate equipment specifications on the effectiveness of proposed technological solutions. The LCI also addressed the varied load balance requirements, accounting for the allocation of power supplied from solar and wind energy sources for bitcoin mining and green hydrogen production in the conventional mitigation framework and total available power from grid electricity supply and green hydrogen–based power generation, which is crucial in the negative mitigation framework. Moreover, detailed operational parameters that affected the LCI calculation, including energy consumption patterns, such as the cooling requirements for the mining equipment, and the performance of auxiliary equipment, such as heat pumps, have been added in *SI Appendix*, Table S2. In addition to the equipment associated with various technological solutions, the LCI also encompassed the dynamic aspects of bitcoin mining operations, such as the network difficulty and block reward essential to calculate the total bitcoins that can be mined. Based on the obtained LCI data, the global warming potential (GWP) indicator over 100 y (GWP100) extracted from the Intergovernmental Panel on Climate Change 2021 life cycle impact assessment method is used to evaluate the impact on climate change. In addition to climate change, the ReCiPe midpoint indicators are used to evaluate the full-spectrum environmental performance of the proposed technological solutions. The specific characterization factors for particulate matter formation, ozone depletion, ionizing radiation, photochemical oxidant formation (human health), human carcinogenic toxicity, human noncarcinogenic toxicity, climate change, water depletion, freshwater ecotoxicity, freshwater eutrophication, terrestrial ecotoxicity, terrestrial acidification, agricultural land occupation, marine ecotoxicity, marine eutrophication, photochemical oxidant formation (ecosystems), fossil resource scarcity, and metal depletion are extracted from the ecoinvent v3.9 database (70) (Fig. 1).

## Results

### Multipronged Strategy for Climate Change Mitigation.

**Fig. 2.**
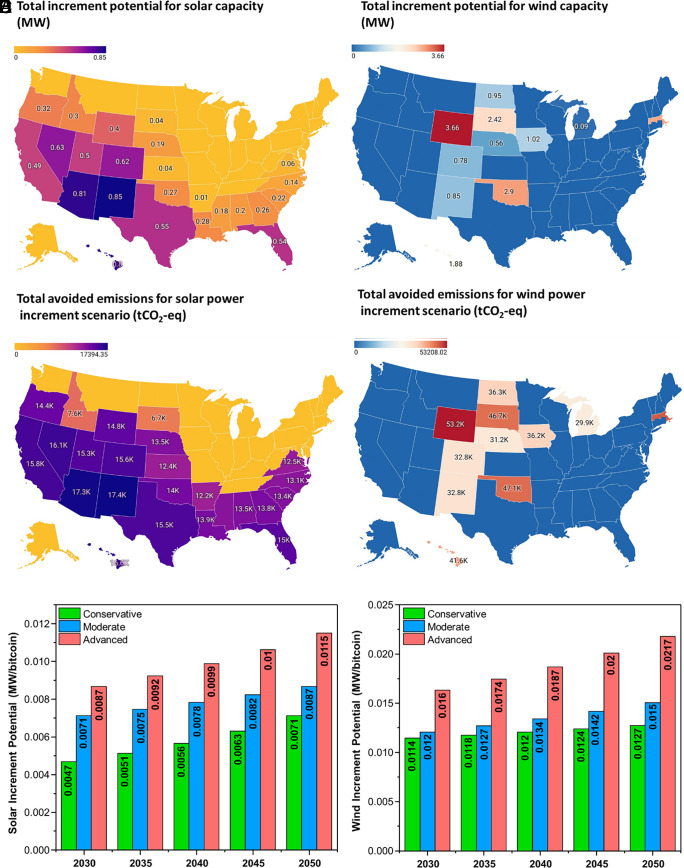
Conventional Mitigation Potential for combined utilization of green hydrogen infrastructure and crypto-operations. (*A*) Total increment potential for solar capacity (MW) in different US states based on initial solar capacity with a direct area equivalent to the generation-weighted average land use of solar PV projects in the United States. (*B*) Total increment potential for wind capacity (MW) in different US states based on an initial wind capacity of 5 MW. (*C*) Total avoided emissions (tCO_2_-eq) based on traditional hydrogen production and grid-powered crypto-operations for solar capacity increment scenario. (*D*) Total avoided emissions (tCO_2_-eq) based on traditional hydrogen production and grid-powered crypto-operations for wind capacity increment scenario. (*E*) Solar power capacity increment for New Mexico utilizing the economic effectiveness of the multipronged strategy under different projection scenarios. (*F*) Wind power capacity increment for Wyoming utilizing the economic effectiveness of the multipronged strategy under different projection scenarios.

This work explores the potential of using green hydrogen and cryptocurrency operations in combination with solar and wind power installations as a viable technological solution for conventional climate change mitigation. Our analysis involves supplying the available power from solar and wind energy systems to a green hydrogen and cryptocurrency mining infrastructure with the economic potential used for expansion of renewable power installations. The initial solar capacity used in our investigation is equivalent to the generation-weighted average land use of solar photovoltaic (PV) projects in the United States, as reported by previous studies (71). Fig. 2*A* presents the findings, which suggest that states like New Mexico have the highest potential for incrementing solar power capacities (0.85 MW), corresponding to the initial investment capacity. Similarly, Fig. 2*B* examines the potential for wind energy systems to act as a mediator for conventional mitigation through bitcoin and green hydrogen. The analysis indicates that while states such as Wyoming (3.66 MW) have a significant potential for incrementing wind energy capacity, some states do not provide wind energy as a feasible alternative to promote conventional mitigation through bitcoin and green hydrogen.

The current practice of using grid-powered operations for bitcoin mining brings about a staggering carbon footprint (36–38, 72, 73), a fact that also applies to global hydrogen production, where 98% of the demand is fulfilled through fossil-heavy production processes such as steam methane reforming and coal gasification (54, 74–76). However, the proposed multipronged strategy seeks to combine bitcoin and green hydrogen production by taking advantage of available renewable energy. For instance, a solar or wind power facility could quench the demand for bitcoin or green hydrogen, thereby avoiding substantial carbon emissions. The correlation between the utilization of renewable power for bitcoin and green hydrogen production and avoided emissions is presented in Fig. 2 *C* and *D* for different US states. States that peak for the highest avoided emissions, such as New Mexico, also emerge as hotspots for potential renewable power development. Similarly, among the wind energy systems, Wyoming trumps the race for avoided emissions among the feasible alternatives. The amount of avoided emissions depends on the type of renewable power generation and the location, a factor that warrants the consideration of carbon credits in the analysis.

The feasibility of the proposed multipronged strategy is influenced by several factors, including the location of the renewable facility and the required capital investment. Since previous studies have demonstrated a reduction in capital intensity for renewable energy installations (48, 77, 78), our analysis incorporates diverse future scenarios utilizing bitcoin and green hydrogen to improve solar and wind energy capacity. Since New Mexico and Wyoming have the highest increment capacity under base case evaluation, Fig. 2 *E* and *F* depicts the potential to increase solar and wind energy capacity under differing projection scenarios. Notably, the most advanced projection scenario indicates that every bitcoin mined in New Mexico through solar power can enhance solar capacity increment by 149% up to 2050. Similarly, using wind energy to mine bitcoin in Wyoming can improve its efficacy by 140%. Even under the most conservative projection scenarios, this strategy facilitates each bitcoin to enhance its economic potential for increment in renewable capacity by 54% and 40% in New Mexico and Wyoming, respectively.

**Fig. 3.**
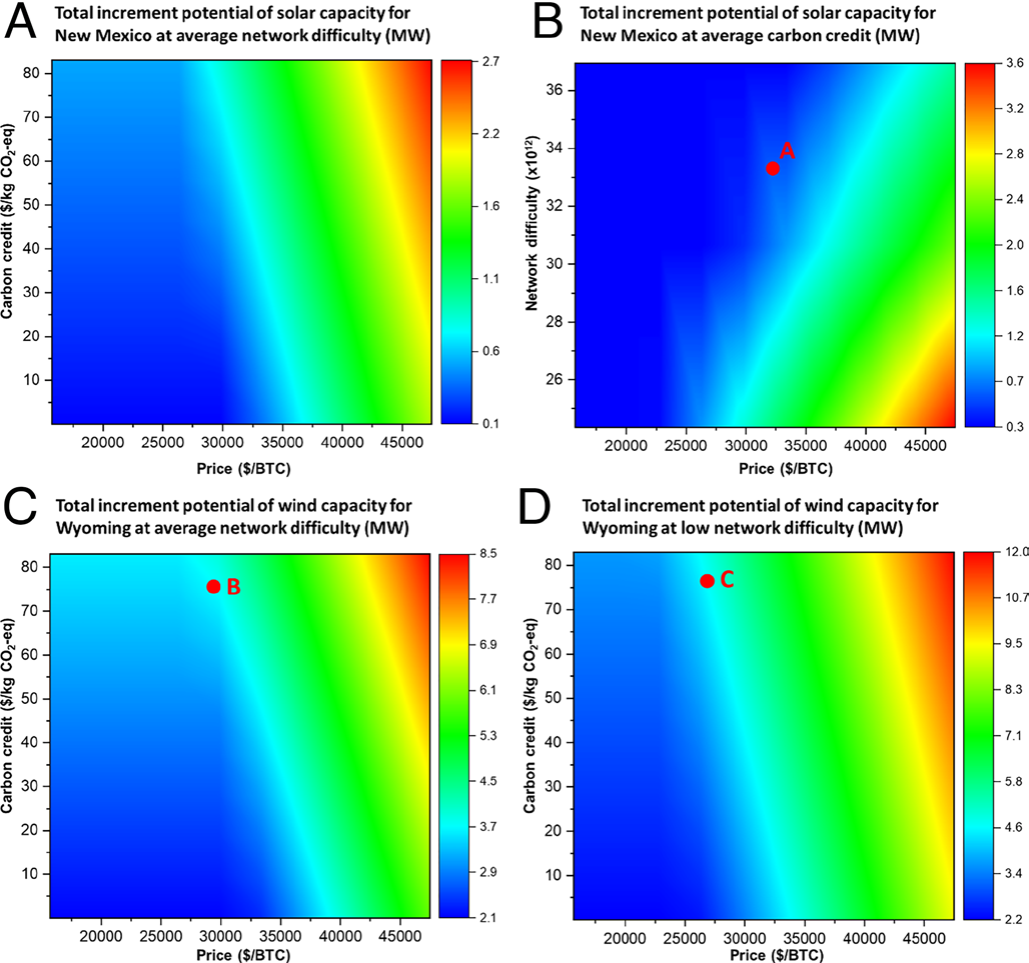
Tackling the uncertainty of crypto-operations to advance conventional climate change mitigation strategies. (*A*) Total solar power capacity increment potential for New Mexico at average network difficulty with varying selling prices and carbon credits. (*B*) Total solar power capacity increment potential for New Mexico at average carbon credit for avoided emissions with varying selling prices and network difficulty. Point “A” depicts that at higher network difficulties with comparatively lower bitcoin prices, the majority of available power gets utilized in green hydrogen production. (*C*) Total wind power capacity increment potential for Wyoming at average network difficulty with varying selling prices and carbon credits. Point “B” depicts that when bitcoin prices are lower, it becomes imperative to enable high carbon credits to support the wind power increment potential of the proposed framework. (*D*) Total wind power capacity increment potential for Wyoming at low network difficulty with varying selling prices and carbon credits. Point “C” depicts a similar trend to point “B” with a relatively shifted contour.

The success of the proposed technological solution is highly contingent upon the fluctuating conditions of the bitcoin network. Specifically, the average network difficulty and selling price are crucial in determining its performance. The network difficulty is a measure used to determine how hard it is for miners to add a new block to the blockchain (79–81). A higher network difficulty implies that it would take more computational power to mine the bitcoin. In an effort to factor in these uncertainties, we carried out a series of parametric sensitivity analyses, which are presented in Fig. 3. Consequently, our approach has the potential to mitigate carbon emissions associated with bitcoin mining and hydrogen production. In addition to examining the network dynamics, we have also evaluated the impact of carbon credits on profitability. To provide a comparative reference, the analysis considered the range of carbon credits from zero to the maximum value equivalent to recent credits, which aim to incentivize carbon capture and storage technologies (82). Notably, the results of the sensitivity analysis are focused on states with the highest potential to deploy solar and wind energy installations: Fig. 3 *A* and *B* corresponds to results for a solar power installation in New Mexico, while Fig. 3 *C* and *D* illustrates the results for the wind energy system in Wyoming.

### Climate Conundrum: Transfer the Energy Carrier or Drive the Renewable Capacity?

**Fig. 4.**
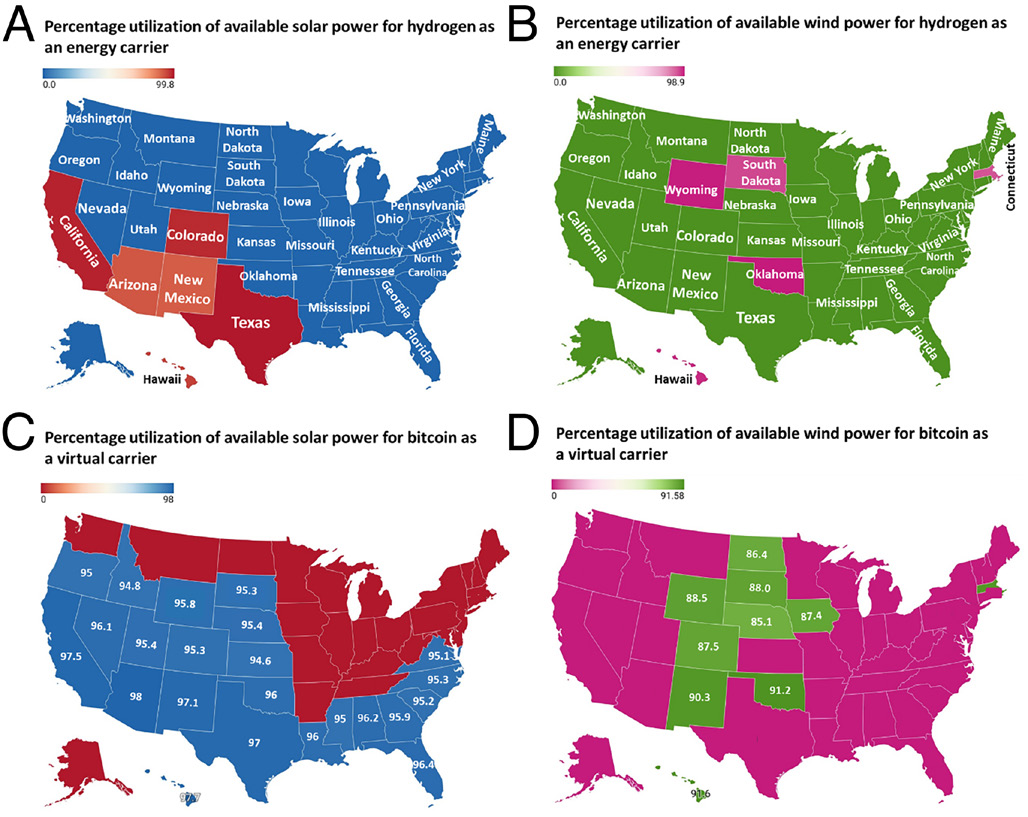
Green hydrogen as a traditional energy carrier in contrast to bitcoin acting as a virtual carrier. (*A*) Percentage utilization of available solar power for hydrogen as an energy carrier in different states. (*B*) Percentage utilization of available wind power for hydrogen as an energy carrier in different states. (*C*) Percentage utilization of available solar power for bitcoin as a virtual carrier in different states. (*D*) Percentage utilization of available wind power for bitcoin as a virtual carrier in different states.

Over the last decade, considerable research and investment have been dedicated to exploring traditional energy carriers, such as green hydrogen, as a means of transferring renewable power from one location to another (83–85). As noted in the previous section, cryptocurrencies like bitcoin can serve to mediate conventional mitigation strategies. Here, we investigate whether the potential of cryptocurrencies to stimulate the development of renewable installations can be used as a viable alternative to traditional energy carriers. The analysis begins with states that have the capability to use solar and wind power facilities to generate green hydrogen as a traditional energy carrier. As illustrated in Fig. 4*A*, certain states, such as New Mexico, have the potential to produce hydrogen energy carriers through their solar power generation facilities. The varying levels of potential between states can be explained by differences in solar irradiation intensity. Similarly, Fig. 4*B* illustrates that wind energy systems in selected states, such as Wyoming, are feasible alternatives to utilizing available power for green hydrogen production. In addition to using the available power for producing a true energy carrier such as green hydrogen, we also investigate whether crypto-operations such as bitcoin can employ its economic potential to enable renewable power generation, thus acting as a virtual energy carrier. By considering the variability in the state-level potential for renewable energy production, we can better assess the potential of leveraging cryptocurrencies in the development of renewable energy infrastructure. We use the levelized costs of renewable power generation to explore how much clean energy can be funded by bitcoin operations in different US states. Fig. 4*C* shows that bitcoin operations can enable solar power generation with maximum utilization of available power at 98%. At the same time, Fig. 4*D* illustrates that bitcoin can facilitate wind power generation with the maximum utilization of available power reaching 92%.

**Fig. 5.**
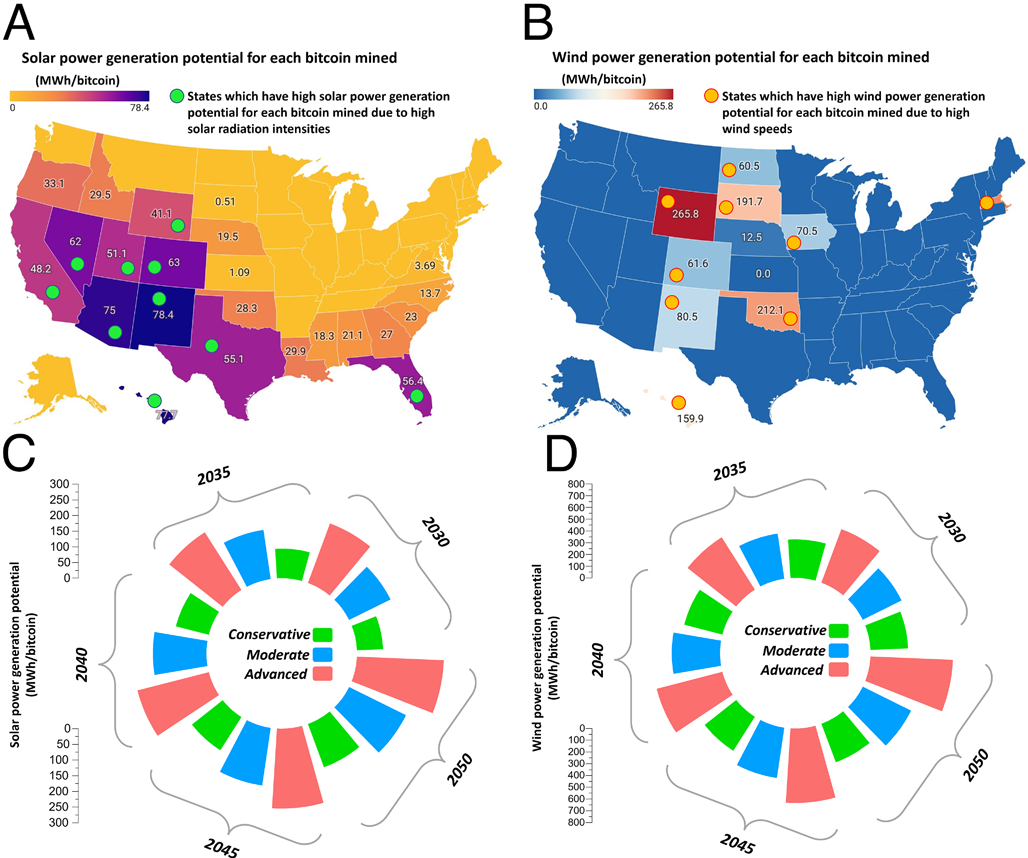
Potential of bitcoin as a virtual carrier across US states under different scenarios. (*A*) Solar power generation potential for each bitcoin mined (MWh/bitcoin) in different US states. The states with green markers (California, Utah, Wyoming, Texas, Florida, Nevada, Arizona, Colorado, New Mexico, and Hawaii) receive higher solar irradiation intensities, so they can generate bitcoins with more economic potential to support future solar installations. (*B*) Wind power generation potential for each bitcoin mined (MWh/bitcoin) in different US states. The states with yellow markers (Wyoming, South Dakota, North Dakota, Iowa, Colorado, New Mexico, Oklahoma, Massachusetts, and Hawaii) receive higher wind speeds and a more favorable distribution, so they can generate bitcoins with more economic potential to support future wind installations. (*C*) Solar power generation potential for each bitcoin mined (MWh/bitcoin) in New Mexico under different projection scenarios. (*D*) Wind power generation potential for each bitcoin mined (MWh/bitcoin) in Wyoming under different projection scenarios.

Fig. 4 *C* and *D* demonstrates how bitcoin operations can serve as a virtual carrier in states using solar and wind power facilities. Using this framework, we can assess the economic potential of each bitcoin mined to promote the development of solar and wind energy infrastructure, as shown in Fig. 5 *A* and *B*, respectively. Fig. 5*A* illustrates that states with higher solar irradiation intensities can generate bitcoins with greater economic potential, enabling the deployment of more solar power infrastructure. As an illustration, New Mexico can use its available solar power resources to mine a bitcoin, allowing 78.4 MWh of solar power generation. Fig. 4*D* indicates that not all states are suitable for bitcoin mining to fund wind power generation. Still, with higher wind speeds, Wyoming can enable each mined bitcoin to support 265.8 MWh of wind energy generation, as illustrated in Fig. 5*B*. These findings underscore the importance of choosing renewable energy facilities in locations with varying geographical advantages, as this can significantly affect the success and economic feasibility of using crypto-operations like bitcoin as virtual energy carriers.

The efficacy of bitcoin operations as a virtual energy carrier depends on the economics of renewable power generation in different US states. However, this potential can be further improved by reducing the levelized cost of clean power generation. We present the projections of the economic potential of bitcoins mined in New Mexico and Wyoming to support clean power generation in Fig. 5 *C* and *D*, respectively. Under an advanced projection scenario, each bitcoin mined using a solar power installation in New Mexico can increase its potential as a virtual energy carrier by 254% through 2050. Similarly, a bitcoin mined with a wind energy system in Wyoming can improve its efficiency as a virtual carrier by 164%. Even under the most conservative projection scenarios corresponding to the levelized cost of solar and wind power generation, the proposed technological solution of bitcoin as a virtual carrier can fund 147.1 and 350.3 MWh of solar and wind energy in New Mexico and Wyoming, respectively. These results suggest that reducing the levelized cost of clean power generation can significantly extend the potential of bitcoin operations as virtual energy carriers, especially in states with favorable renewable power infrastructure development.

### Carbon Offsetting through Carbon-Neutral Operations.

**Fig. 6.**
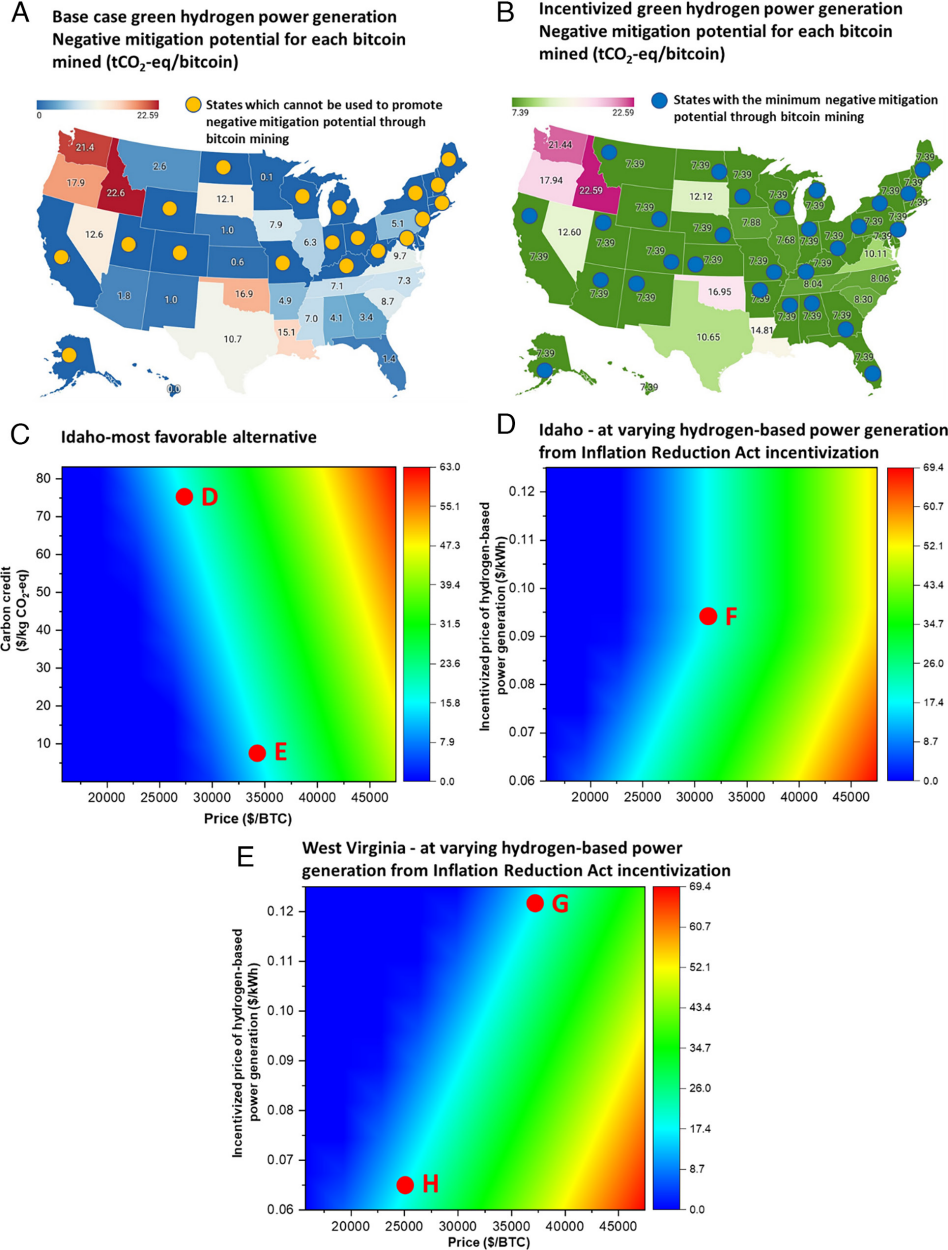
Negative mitigation potential for crypto-operations powered by green hydrogen and grid supply. (*A*) Negative mitigation potential for each bitcoin mined (tCO_2_-eq/bitcoin) under base case green hydrogen power generation. The states with yellow markers cannot be used as feasible alternatives to promote negative mitigation potential through bitcoin mining. (*B*) Negative mitigation potential for each bitcoin mined (tCO_2_-eq/bitcoin) under incentivized green hydrogen power generation. The states with blue markers can attain a minimum negative mitigation potential through bitcoin operations. (*C*) Uncertainty in negative mitigation potential for each bitcoin for Idaho under varying carbon credit and bitcoin selling price. Points “D” and “E” are part of a contour that indicates carbon credits can compensate for lower bitcoin prices in enhancing negative mitigation potential in Idaho. (*D*) Uncertainty in negative mitigation potential for each bitcoin for Idaho under varying degrees of cost reduction for green hydrogen–based power generation due to the IRA and the selling price of bitcoin. Point “F” depicts that even states like Idaho, which have greener electricity mixes, can benefit from higher degrees of incentivization where tax credits low prices for green hydrogen–based power generation (<$0.095/kWh). (*E*) Uncertainty in negative mitigation potential for each bitcoin for West Virginia under varying degrees of cost reduction for green hydrogen–based power generation due to the IRA and selling price of bitcoin. Points “G” and “H” depict a contour that illustrates a higher degree of incentivization for green hydrogen–based power generation reduces the minimum bitcoin price required for mining operations, enabling fossil-heavy state grids to enhance the negative mitigation capacity.

In the current practice, since the bitcoin industry relies heavily on grid electricity, it results in a significant carbon footprint. While negative mitigation technologies like DAC can offset this impact, their cost-effectiveness remains a significant obstacle. Accordingly, we introduce an alternative technological solution to promoting carbon offsetting capacity by investigating whether coupled crypto-operations and green hydrogen–based power generation can facilitate negative mitigation strategies. In this study, we first assess the economic potential of bitcoin mined in different US states to capture CO_2_ emissions using the levelized cost of carbon capture technologies, as depicted in Fig. 6*A*. Results show that some states, such as Idaho, can mine bitcoin and fund the capture of 22.6 tCO_2_-eq, given the high contribution of renewable energy sources in their grid electricity mix and economically favorable retail electricity prices. Conversely, some states indicated with yellow markers in Fig. 6*A* are not feasible alternatives to enhance negative mitigation potential through bitcoin. This trend can be attributed to the high operation costs due to electricity prices, the increased investment required in DAC technologies to achieve carbon neutrality due to the high contribution of fossil-energy sources, and prices from green hydrogen–based power generation. Moreover, we present the economic potential of bitcoin mined under carbon-neutral conditions in different US states with power inputs from the grid and IRA-incentivized green hydrogen–based power generation, as illustrated in Fig. 6*B*. States with greener electricity mix observe no change in their potential for enhancing negative mitigation capacity.

Similar to the case of conventional mitigation framework, it is imperative to investigate how the uncertainty in the network parameters, such as the bitcoin price and the carbon credits issued for the avoided emissions, affect the economic potential of a negative mitigation framework. To analyze this, we first conduct a parametric sensitivity analysis for Idaho, a state that does not depend on incentivizing green hydrogen power generation. As shown in Fig. 6*C*, high bitcoin prices and support from carbon credits can drive up the economic potential of bitcoins mined to facilitate carbon capture capacity. The contour constituting the points “D” and “E” indicates that the issued carbon credits can compensate for a bump in the prices for the carbon-neutral mined bitcoin. However, the degree of incentivization for green hydrogen power generation is also a crucial factor. As depicted in Fig. 6 *D* and *E*, we investigate varying combinations of bitcoin prices and incentivized power generation prices. For instance, Idaho experiences a turning point at $0.095/kWh (point F in Fig. 6*D*), indicating that high tax credits can positively impact the negative mitigation potential of such states, even those with a greener electricity mix. As the degree of incentivization increases, the minimum bitcoin price required for mining operations to achieve carbon neutrality lowers. This reduction in the required bitcoin price, as illustrated by contour points G and H in Fig. 6*E*, enables regions with fossil-dependent grids, such as West Virginia, to increase their negative mitigation capacity more effectively. Point G represents a scenario with lower incentivization, and thus, a higher bitcoin price threshold for the same mitigation potential. In contrast, point H illustrates that increased incentivization significantly reduces the bitcoin price needed to attain equivalent mitigation capacity. This distinction underscores the economic feasibility and potential of cryptocurrency operations to advance the capacity of the negative mitigation framework.

### Environmental Impacts of Proposed Technological Solutions.

**Fig. 7.**
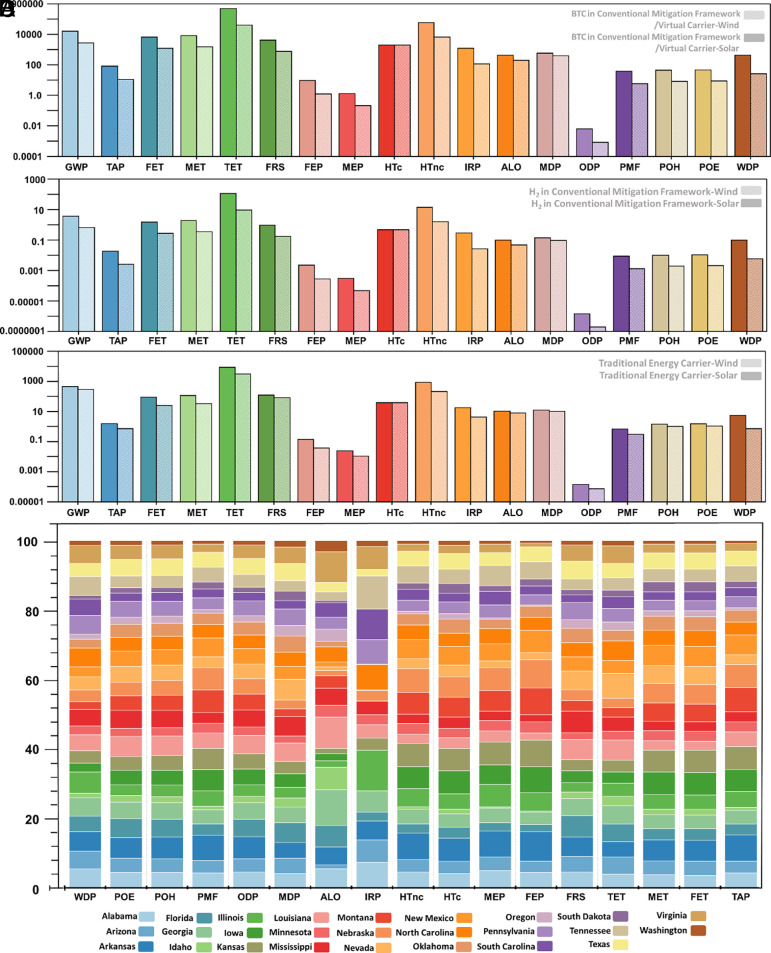
(*A*) Life cycle environmental impacts for each bitcoin mined based on solar and wind energy utilization in the conventional mitigation framework or its applications as a virtual energy carrier. (*B*) Life cycle environmental impacts for each kg of green hydrogen produced based on solar and wind energy utilization in the conventional mitigation framework. (*C*) Life cycle environmental impacts for each MWh of energy supplied using hydrogen as a traditional energy carrier produced using solar and wind energy sources. (*D*) Comparison of life cycle environmental impacts for each bitcoin mined in the negative mitigation framework in different US states. Acronyms: particulate matter formation (PMF); ozone depletion (ODP); ionizing radiation (IRP); photochemical oxidant formation (human health) (POH); human carcinogenic toxicity (HTc); human noncarcinogenic toxicity (HTnc); global warming potential (GWP); water depletion (WDP); freshwater ecotoxicity (FET); freshwater eutrophication (FEP); terrestrial ecotoxicity (TET); terrestrial acidification (TAP); agricultural land occupation (ALO); marine ecotoxicity (MET); marine eutrophication (MEP); photochemical oxidant formation (ecosystems) (POE); fossil resource scarcity (FRS); metal depletion (MDP).

Building upon the analysis of the conventional mitigation framework for climate change and the utilization of traditional and virtual energy carriers using green hydrogen and crypto-operations, we further investigate the LCA results, as illustrated in Fig. 7 *A* and *B*. These findings highlight the distinct environmental impacts beyond avoided emissions while employing solar and wind energy in the technological solutions incorporating bitcoin mining and green hydrogen production. For instance, based on the wind energy utilization in the proposed frameworks, bitcoin mining can attain an 86.4% reduction in terrestrial acidification and an 81.6% reduction in freshwater ecotoxicity compared to solar energy. These substantial decreases underscore wind energy’s lower ecological impact and align with the findings from the analysis, which identifies significant economic potential for wind energy capacity increment. Along similar lines, it is important to note that although solar power utilization also leads to considerably more effects in other impact categories, such as freshwater eutrophication and water depletion, some states can generate significantly high economic potential based on solar power utilization. Furthermore, Fig. 7*C* depicts the life cycle environmental impacts for each MWh of energy supplied using hydrogen as a traditional energy carrier, produced from solar and wind sources. Similar to the case of other technological solutions, using hydrogen as a traditional energy carrier also depicts distinct footprints across various environmental impact categories based on the utilized energy sources. However, while using hydrogen as a traditional carrier with solar and wind energy sources, the percentage difference in the environmental impact categories decreases due to equal contribution from the transportation phase in both cases. In assessing the environmental impacts of bitcoin and green hydrogen production through solar and wind energy utilization in the proposed technological solutions, it is crucial to contextualize the findings within the renewable energy potential across different states. While using wind energy presents a lower impact in certain environmental impact categories along with considerable economic potential in states like Wyoming, it does not diminish the critical role that solar energy can play, especially in states with high solar irradiance, such as New Mexico. These findings necessitate a comprehensive understanding of each state's energy profile, where solar and wind energy sources offer varying degrees of economic potential and the associated environmental impacts. Therefore, the implementation of technological solutions requires a strategic decision based on economic potential, regional characteristics, resource availability, and environmental impact considerations.

In the further investigation of the LCA results, we assess the environmental impact of bitcoin mining across the US states in the negative mitigation framework, as illustrated in Fig. 7*D*. The analysis underscores the correlation between the electricity mix and environmental impacts for each bitcoin mined in the negative mitigation framework across all categories. States with a heavy reliance on fossil energy sources, such as coal and natural gas, in the grid power supply to the proposed framework show increased impacts on resource depletion, human health, and ecosystem quality. On the other hand, states leveraging a higher share of renewable energy sources depict lesser environmental impacts, reinforcing the positive role of renewables in reducing the environmental impact of bitcoin mining. In the case of fossil resource scarcity and metal depletion, the findings reflect the prominent role of regional power supply to mining operations in the negative mitigation framework. For instance, states with a high dependence on natural gas–based power generation, such as Mississippi and Florida, correspond to the highest values in fossil resource scarcity for each bitcoin mined. Similarly, in the water depletion category, Illinois was observed to have the highest impact. This trend can be attributed to certain energy production methods, particularly those involving nuclear power and thermal power plants using fossil fuels, which have higher water footprints compared to renewable sources like wind or solar.

The trends in freshwater and marine eutrophication indicate that states such as Nebraska and Arkansas, which have a major contribution from coal in the electricity mix, have the highest impact in these categories. Moreover, among the states that utilized the grid power supply in the negative mitigation framework, Idaho and Oregon can attain up to 97.1% and 95.2% reduction in these environmental impact categories for each bitcoin mined due to considerably high contributions from clean energy sources, such as hydropower in this case. Similarly, the environmental impact categories that affect human health, such as human toxicity (carcinogenic and noncarcinogenic), ionizing radiation, and particulate matter formation, show significant variations across states. For instance, in the case of human toxicity and particulate matter formation, apart from Nebraska and Arkansas, states such as Montana and Iowa have a considerable impact in these categories due to the utilization of a fossil-dependent energy supply mix in the negative mitigation framework. However, the relative impacts of ionizing radiation can be attributed to the reliance on nuclear power. Accordingly, due to the considerable role of nuclear power in Illinois and South Carolina, each bitcoin mined here based on grid power supply in the negative mitigation framework has a comparatively higher impact in this category. These findings highlight the imperative for a more enhanced role of green hydrogen–based power generation as a displacement for grid power supply in the negative mitigation framework. By shifting toward green hydrogen, which has a lower environmental impact than traditional fossil fuels, states can significantly mitigate the environmental impacts associated with each bitcoin mined. This shift would reduce the overall environmental footprint of bitcoin mining and promote sustainable practices in crypto-operations across different regions.

## Discussion

Over the previous decade, two notable global phenomena have emerged: the growing endorsement of green hydrogen as a key component in the worldwide decarbonization initiatives (20, 23, 27) and the surging interest in the crypto industry (86), despite its negative repercussions on climate change (36–39, 87). The proposed technological solution couples the green hydrogen infrastructure with crypto mining operations powered by solar and wind energy systems in different US states that can propel grid decarbonization. In contrast to criticism that views crypto-operations solely as a contributor to global carbon debt, this study suggests the potential for appropriate policy interventions that could bolster the conventional mitigation frameworks. For example, the systems-level analysis reveals that tapping into the synergistic potential of green hydrogen and bitcoin can facilitate capacity growth of up to 25.5% for solar power and 73.2% for wind power. Similarly, technical innovation can improve the cost-competitiveness of clean energy technologies and green hydrogen infrastructure. Thus, the future scenarios considered in the study depict that the multipronged strategy of green hydrogen and crypto-operations could benefit from leveraging technological advancements to lower the cost of renewable power installation, promoting a low-carbon future. The findings also show that government incentives like carbon credits for clean bitcoin mining and hydrogen production can improve the effectiveness of the proposed strategy for solar and wind power capacity increment. This approach of utilizing economic benefits from this dynamic duo of green hydrogen and crypto could help attract investment to expedite the transition to a cleaner energy matrix, widening its accessibility to the general public.

While evaluating the potential of cryptocurrencies to promote conventional mitigation framework, it is equally important to analyze whether policymakers can utilize crypto-operations to enhance renewable energy penetration at different locations as an effective alternative to traditional energy carriers. The results indicate that states like New Mexico and Wyoming can generate bitcoins with the economic potential to power 78.4 MWh and 265.8 MWh of solar and wind energy, respectively. Similar to the case of conventional mitigation frameworks, technological advancements reducing the cost of location-specific renewable energy generation can improve the performance of virtual carriers. The analysis shows that bitcoin as a virtual carrier can enhance its effectiveness with a projected 254% improvement for solar and 164% for wind energy by 2050. Therefore, varying types of location-specific renewable power facilities can be developed to increase renewable penetration in the grid against accommodating the equipment energy losses while generating the traditional carriers such as hydrogen and extracting the energy from it. This strategy also avoids the emissions due to the carrier’s transport, thus attaining the advantage of renewable energy utilization. Moreover, virtual carriers can contribute to the growth of the local economy by creating employment opportunities in the renewable energy sectors at different locations. In summary, virtual energy carriers can reduce the carbon footprint of energy production and transportation through location-specific renewable energy generation and facilitate climate change mitigation.

As for the influence on negative mitigation framework, this study highlights the potential of crypto-operations to advance the carbon offsetting capacity. The proposed carbon-neutral mining based on the currently prevalent grid supply in conjunction with green hydrogen power generation opens an opportunity to neutralize the climate impacts of crypto-operations. Correspondingly, using the monetary potential from these mining operations to facilitate negative emission technologies provides an added advantage to reduce the climate impact of fossil-based power generation. The findings of the study show that legislative actions like the IRA, which offers tax credits for green hydrogen generation, can enable carbon-neutral bitcoin operations in all US states toward significant carbon reduction. The incentive could also be economic benefits for CO_2_ captured and stored or carbon credits for the avoided emissions due to carbon-neutral mining. State-specific climate incentivization can also be an effective strategy to further strengthen the negative mitigation framework. For example, some states like West Virginia, which have a fossil-dominant grid, can drop the minimum average bitcoin price to promote negative mitigation by 42%, corresponding to a reduction in the incentivized cost of green hydrogen power supply from $125/MWh to $60/MWh. Thus, it is safe to say that adopting legislative actions to promote green hydrogen power generation and supporting states with low fossil fuel dependence will decarbonize crypto-operations and strengthen the negative mitigation framework.

This work utilizes the well-established LCA methodology to evaluate the environmental impacts of proposed technological solutions which incorporate bitcoin mining and green hydrogen production, taking into account various impact categories beyond climate change. The findings reveal distinct environmental footprints for solar and wind energy utilization within the conventional mitigation framework that can be used to enhance renewable energy deployment. For instance, while solar power can be used to harness significant economic potential from the proposed framework, particularly in states with high solar irradiance, it is associated with considerable impacts in categories such as freshwater ecotoxicity, marine ecotoxicity, terrestrial acidification, etc. On the other hand, deploying wind energy within the same framework holds the potential for considerable economic benefits, but it is associated with comparable impacts to solar energy utilization in human toxicity. The results suggest that the proposed framework can generate substantial economic potential using bitcoin mining and green hydrogen production powered through solar and wind energy sources, which can be used to enhance climate mitigation efforts; however, it may introduce shifts in environmental burdens due to the multifaceted nature of environmental impacts associated with renewable energy sources.

Similarly, we evaluate the environmental impacts associated with the negative mitigation framework. The findings highlight that the state electricity mix, along with the proportion of grid supply complemented with the green hydrogen–based power generation, influences the shift in environmental burden across various impact categories. For instance, states employing a significant percentage of renewable sources in their electricity mix exhibit lower impacts in categories such as fossil resource scarcity, particulate matter formation, etc. The significant dependence on fossil energy sources in the negative mitigation framework also increases the impact on human health through enhanced effects in categories such as human toxicity and photochemical oxidant formation. However, the transition to higher reliance on green hydrogen–based power generation along with grid electricity supply can lead to shifts in environmental burdens across freshwater ecotoxicity and marine ecotoxicity. Even among clean energy sources, increased contribution from nuclear energy in the energy supply mix to the negative mitigation framework increases the ionizing radiation potential associated with each bitcoin mined. Therefore, in addition to the economic and operational considerations, the detailed environmental perspectives for the considered technological solutions necessitate a holistic approach to their deployment. Specifically, regional strategies that incorporate ecological sensitivities, human health impacts, and resource depletion should be used to implement climate change mitigation efforts using the proposed technological solutions.

The technological solutions proposed in this work, based on integrating green hydrogen production and bitcoin mining within different frameworks, hold potential for application in various countries. Specifically, countries such as China, Kazakhstan, Russia, Canada, Brazil, etc., have substantial contributions to the computational power in the bitcoin network (88), along with considerable renewable energy resources that can be utilized in the proposed technological solutions. For instance, solar and wind energy resources are expected to play a critical role in China's efforts in energy transition (89, 90), positioning China as a suitable location for the conventional mitigation framework and virtual energy carriers. Similarly, Kazakhstan’s wind energy potential presents opportunities for incorporating cryptocurrency operations with clean energy sources (91). While renewable energy sources have been slow to penetrate the Russian energy market (92), cost-competitive development in the solar PV and wind energy sectors in Russia (93) can be aligned with crypto-operations and green hydrogen production. Along with the prospects of bitcoin mining, green hydrogen production has also gained global momentum. Countries such as Australia and the European Union member states are making strides in green hydrogen projects leveraging their renewable energy capacities (94). Thus, the integration of green hydrogen production in the proposed frameworks enhances its applicability across different regions with specific energy landscapes. Moreover, countries such as Brazil and Canada, which have a substantial contribution from clean energy sources in their electricity mix, such as hydropower in this case (95, 96), hold the potential to utilize crypto-operations in the investigated negative mitigation framework.

Policy and regulatory frameworks can strengthen the adoption of technological solutions in the global context. The findings highlight that incorporating renewable energy sources with bitcoin mining and green hydrogen production has significant implications for climate change mitigation. Therefore, in the case of countries with a favorable stance toward cryptocurrency operations along with heavy reliance on fossil fuels, this approach allows the use of economic benefits from crypto-operations to diversify the energy landscape with renewable energy penetration. By adopting these technologies, countries can attain substantial strides in emissions abatement, contributing to global efforts against climate change. However, it is crucial to recognize that each region is associated with challenges, including economic constraints, technological readiness, and infrastructure deployment needs. Addressing these challenges requires tailored strategies incorporating regional specificities to effectively implement the proposed technological solutions in the global context.

## Conclusion

In this work, we investigated technological solutions incorporating crypto-operations and green hydrogen production to bolster climate mitigation efforts through enhanced deployment of renewable infrastructure and carbon offsetting mechanisms. The multipronged strategy explored the potential of utilizing bitcoin mining coupled with green hydrogen infrastructure in combination with solar and wind power installations to enhance conventional climate change mitigation. In addition, the exploration of cryptocurrencies as virtual energy carriers evaluated the intrinsic economic value of bitcoin mining operations to enable renewable power generation, thus allowing crypto-operations to act as virtual energy carriers. The findings revealed that the effectiveness of the proposed technological solutions varied significantly across different US states, reflecting the diverse renewable energy potential, and underscored the importance of a tailored approach in implementing the conventional mitigation framework and the use of crypto-operations as virtual energy carriers. The strategic decision-making included the state-specific renewable energy potential, economic feasibility, and the associated environmental impacts. The analysis extended beyond conventional climate change mitigation and assessed the economic potential of bitcoin mining in facilitating carbon capture based on grid supply and green hydrogen–based power generation. In the proposed negative mitigation framework, some states exhibited a significant capacity for bitcoin-enabled CO_2_ capture due to favorable renewable energy contributions, while others faced challenges due to higher operational costs and dependency on fossil energy sources. The efficiency of the negative mitigation framework also varied due to the percentage contribution of green hydrogen–based power generation in the total power supply, highlighting the need for region-specific strategies and robust policy development to utilize the negative mitigation potential of crypto-operations. Therefore, the proposed technological solutions offered a path toward facilitating climate change mitigation efforts, leveraging the economic potential of crypto-operations and green hydrogen while incorporating the associated environmental impacts.

## Supplementary Material

Appendix 01 (PDF)

## Data Availability

All study data are included in the article and/or *SI Appendix*.
