# Supplementary material for: Climate sustainability through a dynamic duo: Green hydrogen and crypto driving energy transition and decarbonization
Source: Proc Natl Acad Sci U S A. 2024 Mar 25;121(14):e2313911121. doi: 10.1073/pnas.2313911121 (PMC10998610; doi:10.1073/pnas.2313911121)
Supplement: Supplementary file 1 — Appendix 01 (PDF) [file pnas.2313911121.sapp.pdf]

PNAS

Supporting Information for

Climate Sustainability through a Dynamic Duo: Green Hydrogen and Crypto Driving Energy Transition and Decarbonization

Apoorv Lal, Fengqi You

Fengqi You
Email: fengqi.you@cornell.edu

This file includes:

Supporting text
Figures S1-S6
Table S1-S3

Supporting Information Text

This supplementary text outlines the detailed methodology used to evaluate the potential of bitcoin and green hydrogen as a dynamic duo to support the varying climate change mitigation frameworks. The conventional mitigation framework relies on enhanced renewable energy penetration to mitigate fossil-based GHG emissions. This work investigates how the combined operation of bitcoin mining and green hydrogen production powered through renewable energy sources can increase capital investment in the conventional mitigation framework. As an extension of the conventional mitigation framework, we also examine the potential of utilizing crypto operations, such as bitcoin, as virtual energy carriers that can leverage their monetary value for renewable power generation in diverse settings. The adoption of negative mitigation technologies holds the potential for advancing decarbonization efforts in multiple sectors by implementing carbon offsetting that effectively supports the conventional mitigation framework. Firstly, Section **S1** presents the detailed problem statement illustrating the key input data, model outputs, and major decision variables. The optimization modeling framework devised to investigate the potential of green hydrogen and bitcoin to drive the conventional mitigation framework based on utilizing solar and wind power facilities across US states is depicted in Section **S2**. Subsequently, Section **S3** discusses the equations utilized for the comparative analysis between green hydrogen as a traditional energy carrier and bitcoin as a virtual carrier. Section **S4** shows the optimization modeling framework to investigate the potential of grid-powered crypto operations combined with the green hydrogen power supply to enhance the carbon offsetting capacity. Section **S5** describes the notation employed in the proposed model formulation of different parts of the study. Section **S6** presents the list of input parameters used in the study. Lastly, we conduct sensitivity analyses to investigate the impact of equipment specifications on the effectiveness of the proposed technological solutions in Section **S7**.

S1. Problem Statement

In this work, we investigate technological solutions incorporating cryptocurrencies and green hydrogen production to complement energy transition and decarbonization. The conventional mitigation framework utilizes a clean power supply instead of a fossil-dominant supply for bitcoin mining and green hydrogen production to enhance capital investment in renewable infrastructure. Specifically, we investigate the conventional mitigation framework based on the development of wind and solar power facilities in different US states to mine bitcoin and produce green hydrogen, utilizing their monetary value to increase the renewable energy capacity. The economic potential to increase renewable capacity is also studied for future scenarios to incorporate the varying trajectories for the increasing cost-competitiveness of renewable power generation. In the second part of the study, we delve into the novel concept of using cryptocurrencies as a virtual energy carrier, including a comparative analysis with green hydrogen as a traditional energy carrier based on wind and solar power supply across US states. Lastly, this work investigates the potential of integrating crypto operations with green hydrogen-based power generation in a negative mitigation framework. This framework entails using grid-power supply in conjunction with green hydrogen power supply for crypto mining, from which the economic potential can be used to enhance the carbon-offsetting capacity using technologies like Direct Air Capture (DAC). The general systems optimization framework used in the study for the proposed technological solutions utilized for a given climate change mitigation framework and respective constraints is presented below.

max	NPV for proposed technological solutions
s.t.	Load balance constraints given in eq. (S1)-(S5), (S33)-(S35), (S53)-(S54), and (S70)-(S71)
	Operational constraints given in eq. (S6)-(S16), (S36)-(S39), (S55)-(S59), and (S72)-(S75)
	Economic evaluation constraints given in eq. (S17)-(S32), (S40)-(S52), (S60)-(S69), and (S76)-(S82)

The key input data in this study include:

- The wind speed and solar irradiation data for variable renewable power supply across different US states.

- The performance metrics of renewable power facilities including characteristics such as cut-in and cut-out speeds for wind turbines and solar panel efficiency.
- The network dynamics for the bitcoin mining operations, such as the bitcoin prices and the network difficulties, along with the geographical distribution of mining computational power.
- The equipment specifications for the process sections in the considered technological solutions, including the mining equipment, heat pumps, electrolyzer, and DAC.
- Economic parameters used in the optimization modeling framework, such as the unit capital costs, operational and maintenance costs, and projections for the levelized cost of energy.
- The emission factors in the considered technological solutions, such as the grid power supply for crypto mining operations and conventional pathways for hydrogen production.

The model outputs include:

- The economic potential that can be generated based on the implementation of the considered technological solutions.
- The total deployment of renewable infrastructure which can be attained based on the conventional mitigation framework incorporating bitcoin mining operations and green hydrogen production.
- The potential for total clean energy which can supplied based on the use of crypto operations as virtual energy carriers.
- The total deployment of carbon capture capacity which can be attained based on the negative mitigation framework incorporating bitcoin mining operations and green hydrogen power supply.

The major decision variables include:

- The load balance between power utilized for bitcoin mining and green hydrogen production and the surplus power generation in the conventional mitigation framework, considering the equipment capacities and constraints.
- The load balance between grid power supply in various states and green hydrogen-based power generation utilized for crypto operations in the negative mitigation framework, considering the equipment capacities and constraints.
- The utilized component of the total available power that can be used for deriving the economic potential from crypto operations as virtual energy carriers.
- The economic implications of the considered technological solutions, such as the revenue generation from bitcoin mining and green hydrogen production, capital and operational expenditure for other process sections such as renewable power infrastructure electrolyzer, and DAC.
- The total avoided emissions that can be attained based on the implementation of the considered technological solutions, considering the emission abatement from grid-powered crypto operations and fossil-based conventional pathways for hydrogen production.

All computational experiments are performed on a DELL OPTIPLEX 7040 desktop with Intel(R) Core (TM) i7-6700 CPU @ 3.40GHz and 32 GB RAM. Specifically, GAMS 40.1.0 is used for coding and solving the optimization problem using CPLEX 22.1.0 as the optimizer, with the optimality gap set to 0.01%. The summary of the computational details of the modeling frameworks for different technological solutions, including the constraints, continuous variables, binary variables, and the solution time, are reported in Table S1.

Table S1. Computation details for the optimization modeling frameworks.

Computational Detail	Conventional Mitigation Framework	Virtual Energy Carrier	Negative Mitigation Framework

Binary variables	153	102	153
Continuous variables	243,882	168,861	243,321
Constraints	318,138	224,604	261,834
CPU time (s)	8.84	5.06	7.17

S2. Conventional Mitigation Powered by Crypto and Green Hydrogen

In this work, we investigate a multi-pronged strategy of cryptocurrencies and green hydrogen production to empower renewable generation capacity. This approach explores the potential of bitcoin and green hydrogen production to enhance the conventional mitigation strategies based on switching the energy source from fossil-dominant to renewable energy sources and using the profitability to enhance the capital investment in renewable infrastructure. We study this based on the development of wind and solar power facilities in different US states to mine bitcoin and produce green hydrogen, thus utilizing their monetary value to increase the renewable energy capacity. The economic potential to increase renewable capacity is also studied for future scenarios to consider the expected decrease in the cost associated with renewable power generation. The process starts with collecting the data for wind speed and solar irradiation for the different states considering a renewable power generation facility with a fixed capacity. Power generated by wind turbines is dependent on the incident wind speed. Furthermore, the wind turbine characteristics are the key players in power generation, including the cut-in and cut-out speeds. A piecewise linear equation was used to calculate the wind turbine output power as a function of incident wind speed, as shown in the equation below (1):

$$Pwind(t) = \begin{cases} Pratedwind, & v_r < v^s(t) < v_{cout} \\ Pratedwind \cdot \dfrac{v^s(t) - v_{cin}}{v_r - v_{cin}}, & v_{cin} < v^s(t) < v_r \;, \qquad \forall t \in T \\ 0, & \text{otherwise} \end{cases} \tag{S1}$$

where *Pwind*(*t*) is the wind power output at time *t*. *Pratedwind* is the rated output of the wind turbine. v_r, v_{cin}, and v_{cout} are the rated wind speed and the cut-in and cut-out wind speeds, respectively. $v^s(t)$ is the wind speed at any given location at time *t*, and *T* refers to the total time intervals for the project life.

On the other hand, for a solar photovoltaic (PV) system, which converts solar radiation into power, the total power available at a given time is a function of incident radiation, the efficiency of the solar panel, and the surface area of the collector panels. It can be represented as a linear function as shown below (1):

$$Psolar(t) = \mu^{PV} \cdot rsolar(t) \cdot S^{PV}, \qquad \forall t \in T \tag{S2}$$

where *Psolar*(*t*) is the output PV power, μ^{PV} is the solar panel efficiency, *rsolar*(*t*) is the incident solar irradiation, and S^{PV} is the panel surface area. The calculated total wind or solar power values can be represented by a general term (*Pavailable*(*t*)), which describes the total available power in different time intervals. The following equation can describe the load balance of the available power:

$$Pavailable(t) = Putilized(t) + Psurplus(t), \qquad \forall t \in T \tag{S3}$$

where *Putilized*(*t*) and *Psurplus*(*t*) represent the utilized and surplus power at different time intervals. The total utilization (*UTL*) for the available energy from the installed wind or solar power facility can be calculated using Eq. (S4) as shown below:

$$UTL = \frac{\sum_{t \in T} Putilized(t)}{\sum_{t \in T} Pavailable(t)} \quad \text{(S4)}$$

Subsequently, the load balance for the utilized power can be described using the distribution among the varying components:

$$Putilized(t) = Pminer(t) + Phpump(t) + Pele(t), \qquad \forall t \in T \quad \text{(S5)}$$

where *Pminer*(*t*), *Phpump*(*t*), and *Pele*(*t*) refer to the power dedicated to the mining equipment, heat pumps, and the green hydrogen infrastructure. However, the total power utilized for cryptocurrencies must be less than the cumulative power capacity of the mining equipment. Thus, using *Nminer* as the number of mining equipment and *Pminermax* as the capacity of individual mining equipment, Eq. (S6) describes the maximum limit to the power dedicated to the crypto-mining process.

$$Nminer \cdot Pminermin \leq Pminer(t) \leq Nminer \cdot Pminermax, \qquad \forall t \in T \quad \text{(S6)}$$

The total heat that must be removed from the mining equipment (*Pheat*(*t*)) can be calculated using the total power consumed by the mining equipment as follows:

$$Pheat(t) = Pminer(t) \cdot hf, \qquad \forall t \in T \quad \text{(S7)}$$

where *hf* is the associated factor for the cooling load in the mining equipment. Considering the coefficient of performance (*COP*) for the heat pumps, the total power consumed by heat pumps can be calculated using Eq. (S8).

$$Pheat(t) = Phpump(t) \cdot COP, \qquad \forall t \in T \quad \text{(S8)}$$

However, there are limits to the total power consumed in heat pumps based on the number of heat pumps (*Nhpump*) utilized and the individual capacities as described in the following equations:

$$Nhpump \cdot phmin \leq Phpump(t) \leq Nhpump \cdot phmax, \qquad \forall t \in T \quad \text{(S9)}$$

where *phmax* and *phmin* refer to the heat pump's maximum and minimum power consumption. Similarly, using the number of electrolyzers (*Nele*), i.e., the total power utilized in the electrolyzer unit, must follow the equation:

$$Nele \cdot pemin \leq Pele(t) \leq Nele \cdot pemax, \qquad \forall t \in T \quad \text{(S10)}$$

where *pemax* and *pemin* represent the electrolyzer's maximum and minimum power consumption levels. The total life cycle emissions corresponding to power generation can be calculated using the characterization factors for renewable energy, i.e., *cfrenew*. Thus, Eq. (S11) describes the total emissions associated with mining bitcoin and producing green hydrogen using the available infrastructure.

$$Emtotal = \sum_{t \in T} \left(cfrenew \cdot Putilized(t) \right) \quad \text{(S11)}$$

Now, based on the distribution of the hash rate for the given cryptocurrency, the emissions corresponding to mining the same number of coins (*Emoriginalcrypto*) under the usual grid power supply can be calculated based on the following equations:

$$Emoriginalcrypto = \sum_{j \in J} cfelec(j) \cdot x(j) \cdot \sum_{t \in T} \frac{R \cdot H(t) \cdot t_m}{D(t) \cdot 2^{32}} \tag{S12}$$

$$\cdot \sum_{j \in J} x(j) = 1 \cdot \tag{S13}$$

where *cfelec*(*j*) and *x*(*j*) represent the characterizations factor for the geographical location and the percentage of miners in that location, respectively. Also, we require the data corresponding to the network difficulty (*D*(*t*)), hashing power (*D*(*t*)) corresponding to the energy utilized in the mining equipment, the reward for the number of bitcoins for each block mined on the blockchain network (R), and the mining time interval (t_m). Mining difficulty indicates how difficult it is to verify a transaction in the network and, thus, add a block in the blockchain to get a given currency as the reward.

Similarly, based on the percentage contribution of different hydrogen production pathways (*y*(*m*)) and their respective characterization factors (*cfprocess*(*m*)), the total emissions avoided due to hydrogen production (*Emoriginalhydrogen*) can be calculated as:

$$Emoriginalhydrogen = \sum_{m \in M} cfprocess(m) \cdot y(m) \cdot \sum_{t \in T} h2(t) \tag{S14}$$

$$\sum_{m \in M} y(m) = 1 \tag{S15}$$

Thus, the total avoided emissions (*Emavoided*) due to the utilization of renewable power for bitcoin and green hydrogen can be calculated using Eq. (S16).

$$Emavoided = Emoriginalcrypto + Emoriginalhydrogen - Emtotal \tag{S16}$$

The economic evaluations start by estimating the total revenue for the project (*REVENUE*), which can be calculated as the summation of the income at different time intervals, represented as follows:

$$REVENUE = \sum_{t \in T} Revcrypto(t) + Rehydrogen(t) \tag{S17}$$

where *Revcrypto*(*t*) and *Revhydrogen*(*t*) represent the revenue generated in different time intervals for mining cryptocurrencies and green hydrogen production for the respective scenarios. The revenue generated from the crypto mining process depends on the price of the currency (SP^{CR}), apart from the number of coins rewarded on adding a new block, hashing power, network difficulty, and the mining time interval, as shown in Eq. (S18).

$$Revcrypto(t) = \frac{SP^{CR} \cdot R \cdot H(t) \cdot t_m}{D(t) \cdot 2^{32}}, \qquad \forall t \in T \tag{S18}$$

Subsequently, based on the power which has been utilized in the electrolyzer units (*Pele*(*t*)), the hydrogen produced (*h2*(*t*)) can be calculated as follows:

$$h2(t) = \frac{Pele(t) \cdot ele}{LHV}, \qquad \forall t \in T \tag{S19}$$

where *ele* and *LHV* represent the efficiency of the electrolyzer and the lower heating value for hydrogen in kWh/kg of H_2. Using the selling price of hydrogen (H_2^{SP}), the revenue generated can be calculated as follows:

$$Revhydrogen(t) = \sum_{t \in T} H_2^{SP} \cdot h2(t), \qquad \forall t \in T \tag{S20}$$

The capital expenditure for the different process components can be calculated using the following equations:

$$Cminer = Nminer \cdot ACminer \cdot repl \tag{S21}$$

$$Chpump = Nhpump \cdot AChpump \tag{S22}$$

$$Crenewable = Nrenewable \cdot ACrenewable \tag{S23}$$

$$Cele = Nele \cdot ACele \tag{S24}$$

where *ACminer*, *AChpump*, *ACrenewable,* and *ACele* refer to the unit capital cost for miners, heat pumps, solar or wind energy investment, and the required green hydrogen infrastructure. Also, *repl* refers to replacing mining equipment based on the total project life. Based on the considered project life and the components used, the total salvage value (*SAL*) can be calculated for all the equipment used using their respective depreciation factors.

$$SALminer = sminer \cdot Cminer \tag{S25}$$

$$SALhpump = shpump \cdot Chpump \tag{S26}$$

$$SALrenewable = srenewable \cdot Crenewable \tag{S27}$$

$$SALele = sele \cdot Cele \tag{S28}$$

where *SALminer*, *SALhpump*, *SALrenewable,* and *SALele* refer to the salvage values for miners, heat pumps, solar or wind energy investment, and the required green hydrogen infrastructure. Also, *sminer*, *shpump*, *srenewable*, and *sele* refer to the corresponding depreciation factors. The operating cost for the renewable facility (*Opexrenewable*) and the heat pumps (*Opexhpump*) can be calculated using Eq. (S29) and Eq. (S30).

$$Opexhpump = \sum_{t \in T} Opexhp(t) = \sum_{t \in T} Phpump(t) \cdot opfachp \tag{S29}$$

$$Opexrenewable = \sum_{t \in T} Opexrenew(t) = \sum_{t \in T} Putilized(t) \cdot opfacrenew \tag{S30}$$

where *opfachp* and *opfacrenew* refer to the summed operational and maintenance cost units for the heat pump and renewable facility, respectively. The operating expenditure for the electrolyzer unit ($OPEXele$) is based on a specific percentage of capital cost for each year (*cfele*).

$$OPEXele = Cele \cdot cfele \cdot NY \tag{S31}$$

Now, based on the NPV from the facility's operation and the unit capital investment for the specific renewable power facility, the incremental capacity of clean power generation (*addrenew*(*k*)), which can be installed is calculated using the Eq. (S32).

$$addrenew(k) = \frac{NPV(k)}{ACrenew(k)} \tag{S32}$$

where "k" refers to the index for the year considered in the base case evaluation or future scenarios $k \in [2030,\ 2035,\ 2040,\ 2045,\ 2050]$.

S3. Virtual and Traditional Energy Carrier

This study considers that a solar or wind power facility produces an energy carrier that can supply electricity at a different location. As a traditional energy carrier, green hydrogen has been chosen in the study. The location of the facility has been considered in all the states in the US. In contrast, the same solar or wind facility in each state has been analyzed to produce the given cryptocurrency. This intrinsic monetary value can be used to increase renewable power generation at the other location, thus allowing crypto operations to act as a virtual energy carrier.

Crypto operations to create energy

Selective equations involving the bitcoin network dynamics from the previously described Eq. (S1) to Eq. (S32) can be used to calculate how much increased renewable energy capacity can be generated. In this case, we consider the profitability of the given bitcoin operation to be used to fund the renewable power generation based on the respective levelized cost. Also, like the previous case of using crypto to increase renewable investments in future scenarios, we consider here $k \in [2030,\ 2035,\ 2040,\ 2045,\ 2050]$ to capture the effect of expected decrement in the levelized cost for renewable power generation. We consider the installation of solar and wind generation facilities in different US states with the governing equations as Eq. (S1) and Eq. (S2), respectively. Similar to Eq. (S3), the following equation shows that some component of the total available power ($Pavailable(t)^{crypto}$) remains utilized in different time intervals ($Psurplus(t)^{crypto}$).

$$Pavailable(t)^{crypto} = Putilized(t)^{crypto} + Psurplus(t)^{crypto}, \qquad \forall t \in T \tag{S33}$$

Based on the utilized power component in different time intervals ($Putilized(t)^{crypto}$), Eq. (S34) shows the distribution between the mining equipment ($Pminer(t)^{crypto}$) and the heat pumps ($Phpump(t)^{crypto}$) for the case of crypto as the virtual energy carrier.

$$Putilized(t)^{crypto} = Pminer(t)^{crypto} + Phpump(t)^{crypto}, \qquad \forall t \in T \tag{S34}$$

The total utilization (UTL^{crypto}) for the available energy from the installed wind or solar generation facility in the case of crypto as the virtual carrier can be calculated using Eq. (S35) as shown below:

$$UTL^{crypto} = \frac{\sum_{t \in T} Putilized(t)^{crypto}}{\sum_{t \in T} Pavailable(t)^{crypto}} \tag{S35}$$

Now, based on the number of miners ($Nminer^{crypto}$) and the heat pumps ($Nhpump^{crypto}$), the power utilized in different components should follow the equations:

$$Nminer^{crypto} \cdot Pminermin \le Pminer(t)^{crypto} \le Nminer^{crypto} \cdot Pminermax, \qquad \forall t \in T \tag{S36}$$

$$Nhpump^{crypto} \cdot phmin \le Phpump(t)^{crypto} \le Nhpump^{crypto} \cdot phmax, \qquad \forall t \in T \tag{S37}$$

Eq. (S38) describes the total life cycle emissions associated with mining bitcoin using the available renewable infrastructure based on the respective characterization factor. For the calculation of avoided emissions, we first use Eq. (S12) and (S13) to get the emissions corresponding to the grid-powered mining operations. Subsequently, we use the following equation to get the avoided emissions using crypto as the virtual carrier.

$$Emtotal^{crypto} = \sum_{t \in T} \left(cfrenew \cdot Putilized(t)^{crypto} \right) \tag{S38}$$

$$Emavoided^{crypto} = Emoriginalcrypto - Emtotal^{crypto} \tag{S39}$$

The economic calculations for crypto as the virtual carrier would depend on the income in different time intervals in Eq. (S39) using the power utilized by the miners.

$$Revcrypto(t)^{crypto} = \frac{SP^{CR} \cdot R \cdot H(t)^{crypto} \cdot t_m}{D(t) \cdot 2^{32}}, \qquad \forall t \in T \tag{S40}$$

Correspondingly, the capital expenditure for using crypto as a virtual carrier depends on the cost of miners and heat pumps as described below:

$$Ctotal^{crypto} = Cminer^{crypto} + Chpump^{crypto} + Crenewable^{crypto} \tag{S41}$$

$$Cminer^{crypto} = Nminer^{crypto} \cdot ACminer \cdot repl \tag{S42}$$

$$Chpump^{crypto} = Nhpump^{crypto} \cdot AChpump \tag{S43}$$

$$Crenewable^{crypto} = Nrenewable^{crypto} \cdot ACrenewable \tag{S44}$$

where $Cminer^{crypto}$, $Chpump^{crypto}$, and $Crenewable^{crypto}$ refer to the capital cost for miners, heat pumps, and solar or wind energy investment for using crypto as the virtual carrier. Based on the depreciation factors described in Eq. (S25)-(S27), the following equations estimate the salvage values:

$$SALtotal^{crypto} = SALminer^{crypto} + SALhpump^{crypto} + SALrenewable^{crypto} \tag{S45}$$

$$SALminer^{crypto} = sminer \cdot Cminer^{crypto} \tag{S46}$$

$$SALhpump^{crypto} = shpump \cdot Chpump^{crypto} \tag{S47}$$

$$SALrenewable^{crypto} = srenewable \cdot Crenewable^{crypto} \tag{S48}$$

where $SALminer^{crypto}$, $SALhpump^{crypto}$, and $SALrenewable^{crypto}$ refer to the salvage values for miners, heat pumps, and solar or wind energy investment for using crypto as the virtual carrier. Also, Eq. (S49)-(S51) describe the equations used to calculate the operating cost for using crypto operations as the virtual energy carrier.

$$Opex^{crypto} = Opexrenewable^{crypto} + Opexhpump^{crypto} \tag{S49}$$

$$Opexhpump^{crypto} = \sum_{t \in T} Opexhp(t)^{crypto} = \sum_{t \in T} Phpump(t)^{crypto} \cdot opfachp \tag{S50}$$

$$Opexrenewable^{crypto} = \sum_{t \in T} Opexrenew(t)^{crypto} = \sum_{t \in T} Putilized(t)^{crypto} \cdot opfacrenew \tag{S51}$$

Lastly, based on the NPV for the facility's operation in the project lifetime and the levelized cost for specific renewable power generation (*lcoe*), the total clean power ($pnew^{crypto}$), which can be funded using the crypto operations based on the location-specific renewable power facility can be calculated as follows:

$$pnew(k)^{crypto} = \frac{NPV(k)^{crypto}}{lcoe(k)} \tag{S52}$$

where "k" refers to the index for the base case evaluation or the year considered in future scenarios $k \in [2030,\ 2035,\ 2040,\ 2045,\ 2050]$.

Green hydrogen carrier for comparative analysis

In order to use hydrogen as the traditional energy carrier alternative, we consider an electrolyzer that takes power as input and produces green hydrogen. Similar to the case of crypto as the virtual carrier Eq. (S1) and Eq. (S2) are used to calculate the available power in different time intervals to produce the green hydrogen carrier. The following equation shows that some component of the total available power ($Pavailable(t)^{hyd}$) remains utilized in different time intervals ($Psurplus(t)^{hyd}$).

$$Pavailable(t)^{hyd} = Pele(t)^{hyd} + Psurplus(t)^{hyd}, \qquad \forall t \in T \tag{S53}$$

Based on the utilized power component in different time intervals, Eq. (S53) shows that some fraction of available power is used in the electrolyzer units in each time interval. The total utilization (UTL^{hyd}) for the available energy from the installed wind or solar generation facility in the case of hydrogen as the traditional carrier can be calculated using Eq. (S54) as shown below:

$$UTL^{hyd} = \frac{\sum_{t \in T} Putilized(t)^{hyd}}{\sum_{t \in T} Pavailable(t)^{hyd}} \tag{S54}$$

Now, based on the number of electrolyzer units ($Nele^{hyd}$), the power utilized in different time intervals should follow the following equation:

$$Nele^{hyd} \cdot pemin \le Pele(t)^{hyd} \le Nele^{hyd} \cdot pemax, \qquad \forall t \in T \tag{S55}$$

Corresponding to the power which has been utilized in the electrolyzer, the hydrogen produced in different time intervals ($h2(t)^{hyd}$) can be calculated using Eq. (S56).

$$h2(t)^{hyd} = \frac{Pele(t)^{hyd} \cdot \eta^{ELE}}{LHV}, \qquad \forall t \in T \tag{S56}$$

Now considering the efficiency of the energy extraction process (*effc*), the total electricity (*etotal*) which can be supplied at the location can be calculated as follows:

$$etotal = \sum_{t \in T} h2(t)^{hyd} \cdot LHV \cdot effc \tag{S57}$$

Eq. (S58) describes the total life cycle emissions associated with using the renewable power facility based on the respective characterization factor to produce green hydrogen. The other emission, in this case, is associated with the transport of the produced hydrogen, which can be calculated using the characterization factor for transporting the produced hydrogen (*cftransport*) and the distance (*d*).

$$Emtotal^{hyd} = \sum_{t \in T} \left(cfrenew \cdot Putilized(t)^{hyd} \right) + \sum_{t \in T} cftransport \cdot h2(t)^{hyd} \cdot d \tag{S58}$$

To calculate avoided emissions ($Emavoided^{hyd}$), we first use Eq. (S14) and (S15) to get the emissions corresponding to global hydrogen production using the contribution of varying pathways. Subsequently, we use the following equation to get the avoided emissions based on using green hydrogen as a traditional energy carrier.

$$Emavoided^{hyd} = Emoriginalhydrogen - Emtotal^{hyd} \tag{S59}$$

The capital expenditure for using hydrogen as a traditional carrier depends on the cost of the electrolyzer and the renewable power installation as described below:

$$Ctotal^{hyd} = Cele^{hyd} + Crenewable^{hyd} \tag{S60}$$

$$Cele^{hyd} = Nele^{hyd} \cdot ACele \tag{S61}$$

$$Crenewable^{hyd} = Nrenewable^{hyd} \cdot ACrenewable \tag{S62}$$

where $Cele^{hyd}$ and $Crenewable^{hyd}$ refer to the capital cost for electrolyzer infrastructure and solar or wind energy investment for using green hydrogen as the traditional carrier. Based on the depreciation factors described in Eq. (S27) and (S28), the following equations estimate the salvage values:

$$SALtotal^{hyd} = SALele^{hyd} + SALrenewable^{crypto} \tag{S63}$$

$$SALele^{hyd} = sele \cdot Cele^{hyd} \tag{S64}$$

$$SALrenewable^{hyd} = srenewable \cdot Crenewable^{hyd} \tag{S65}$$

where $SALele^{hyd}$ and $SALrenewable^{hyd}$ refer to the salvage values for electrolyzer infrastructure and solar or wind energy investment for using green hydrogen as the traditional carrier. Also, Eq. (S66)-(S69) describe the equations used to calculate the operating cost for using green hydrogen as a traditional energy carrier, including the transportation cost of the produced hydrogen ($Opextransport^{hyd}$) based on the total hydrogen production in the project life and the cost per unit mass (*hst*) and distance to be transported.

$$Opex^{hyd} = Opexrenewable^{hyd} + Opexele^{hyd} + Opextransport^{hyd} \tag{S66}$$

$$Opexele^{hyd} = Cele^{hyd} \cdot cfele \cdot NY \tag{S67}$$

$$Opexrenewable^{hyd} = \sum_{t \in T} Opexrenew(t)^{hyd} = \sum_{t \in T} Putilized(t)^{hyd} \cdot opfacrenew \tag{S68}$$

$$Opextransport^{hyd} = \sum_{t \in T} h2(t)^{hyd} \cdot hst \cdot d \tag{S69}$$

S4. Negative Mitigation Powered by Crypto coupled with Green Hydrogen

This section presents the framework utilized to investigate the potential of crypto operations to enhance negative climate change mitigation technology. Direct Air Capture (DAC) technology can be a carbon offsetting alternative with any power generation facility. The extent of utilization of the DAC technology would depend on the net emissions from the process to eventually reach the condition of carbon neutrality. In this work, we investigate the utilization of currently prevalent grid-powered crypto mining under carbon-neutral conditions, the profit from which is eventually utilized to increase the carbon-offsetting capacity for the power sources considered. Apart from the grid supply, this framework also incorporates power generation from green hydrogen, contributing to the total power utilized in either mining or operating the carbon-offsetting technology. In this case, let $Ptotal(t)^{neg}$ represent the power generation where the total load balance of power can be described using Eq. (S70):

$$Ptotal(t)^{neg} = Pminer(t)^{neg} + Phpump(t)^{neg} + Pdac(t)^{neg}, \qquad \forall t \in T \tag{S70}$$

$$Ptotal(t)^{neg} = Pgrid(t)^{neg} + Phydrogen(t)^{neg}, \qquad \forall t \in T \tag{S71}$$

where $Pdac^{neg}(t)$ refers to the power dedicated to the DAC technology. Accordingly, $Pminer(t)^{neg}$ and $Phpump(t)^{neg}$ represent the power utilized in miners and the heat pumps for the negative mitigation framework. The total load balance is also depicted in Eq. (S71), where $Pgrid(t)^{neg}$ and $Phydrogen(t)^{neg}$ represent the power import from the grid and electricity generation using green hydrogen. The equations utilized to calculate the revenue generated, the corresponding cost of the mining equipment and the auxiliary equipment, and the limits to the power utilization in the individual equipment remain the same as described before for the conventional mitigation part and using crypto as the virtual carrier. However, similar to the mining and the auxiliary equipment, there are limits to the total power consumed DAC based on the number of DAC units (*Ndac*) utilized and the individual capacities as described in Eq. (S72), where *dacmax* and *dacmin* refer to the maximum power consumption in the DAC units.

$$Ndac \cdot dacmin \le Pdac\left(t\right)^{neg} \le Ndac \cdot dacmax, \qquad \forall t \in T \tag{S72}$$

Eq. (S73) calculates the total emission for the process ($Emtotal^{neg}$) based on the characterization factor for a particular state electricity grid (*cfgrid*). Now, as per the condition of carbon neutrality (Eq. (S74)), the summation of the amount of CO_2 captured by the DAC ($CO2DAC(t)$) should equal the total emissions in the project life. The amount of CO_2 captured by the DAC depends on the power supplied to run this equipment and the corresponding efficiency for the equipment (*dacfactor*), as depicted in Eq. (S75).

$$Emtotal^{neg} = \sum_{t \in T} Pgrid\left(t\right)^{neg} \cdot cfgrid \tag{S73}$$

$$\sum_{t \in T} CO2DAC\left(t\right) = Emtotal^{neg} \tag{S74}$$

$$CO2DAC\left(t\right) = dacfactor \cdot Pdac\left(t\right)^{neg}, \qquad \forall t \in T \tag{S75}$$

Apart from the initial investment required in the miners and heat pumps as described in previous parts of the study, the capital expenditure (*Cdac*) and the salvage value (*SALdac*) for the DAC units utilized can be calculated using Eq. (S76) and (S77), respectively. Similarly, Eq. (S78) calculates the operating cost (*OPEXdac*) for the DAC units.

$$Cdac = Ndac \cdot ACdac \tag{S76}$$

$$SALdac = sdac \cdot Cdac \tag{S77}$$

$$OPEXdac = cfdac \cdot Cdac \cdot NY \tag{S78}$$

where *ACdac* and *sdac* refer to the unit capital cost and depreciation factor for DAC units. Also, *cfdac* refers to the percentage conversion between capital cost and operating cost for DAC. The other change in the cost components is the operating cost for the grid electricity import ($Opexgrid^{neg}$) and the power generation using green hydrogen ($Opexhyd^{neg}$), which can be calculated using the retail price of electricity (*prgrid*), total grid power import, cost of power from green hydrogen (*phyd*), and green hydrogen power import. Another element in the total operating cost is the storage and transportation cost for the captured CO_2 ($Opexccs^{neg}$) as described by Eq. (S81), where *ctr* and *cst* refer to the cost of transport and storage of captured CO_2, respectively.

$$Opexgrid^{neg} = \sum_{t \in T} Pgrid\left(t\right)^{neg} \cdot prgrid \tag{S79}$$

$$Opexhyd^{neg} = \sum_{t \in T} Phydrogen\left(t\right)^{neg} \cdot phyd \tag{S80}$$

$$Opexccs^{neg} = \sum_{t \in T} CO2DAC\left(t\right) \cdot \left(ctr + cst\right) \tag{S81}$$

Lastly, based on the NPV for the facility's operation in the project lifetime and the levelized cost for capturing the CO_2 (*lcap*), the total amount of CO_2 capture (cap^{neg}), which can be funded using the crypto operations based grid powered operation and green hydrogen power supply can be calculated as follows:

$$cap^{neg} = \frac{NPV^{neg}}{lcap} \tag{S82}$$

S5. Notation

Sets/Indices

J	Set representing the spatial distribution of bitcoin miners
M	Set representing the contribution of different hydrogen production pathways in the global hydrogen demand
T	Set representing the time intervals in the different parts of the study

Variables

addrenew	Total new renewable capacity which can be funded through the multi-pronged approach in the conventional mitigation framework (MW)
cap^{neg}	Total CO_2 capture which can be funded from carbon-neutral bitcoins in the negative mitigation framework (kg CO_2-eq)
Cdac	Total capital expenditure on DAC units in the negative mitigation framework ($)
Cele	Total capital expenditure on the green hydrogen infrastructure in the conventional mitigation framework ($)
$Cele^{hyd}$	Total capital expenditure on the green hydrogen infrastructure for using green hydrogen as a traditional energy carrier ($)
cfelec (*j*)	Characterization factor for the grid electricity for the given geographical location of miner, represented by the index *j* (kgCO_2-eq/kWh)
cfgrid	Characterization factor for the respective state electricity grids in the negative mitigation framework (kgCO_2-eq/kWh)
cfprocess (*m*)	Characterization factor for different hydrogen pathways in meeting the global hydrogen demand, represented by the index *m* (kgCO_2-eq/kg H_2)
Chpump	Total capital expenditure on the auxiliary heat pumps in the conventional mitigation framework ($)
$Chpump^{crypto}$	Total capital expenditure on the auxiliary heat pumps for using crypto as the virtual energy carrier ($)
Cminer	Total capital expenditure on the mining equipment in the conventional mitigation framework ($)
$Cminer^{crypto}$	Total capital expenditure on the mining equipment for using crypto as the virtual energy carrier ($)
CO2DAC(*t*)	Amount of carbon dioxide captured by DAC in different time intervals, represented by the index *t* (kg)
Crenewable	Total capital expenditure on the renewable power installation in the conventional mitigation framework ($)
$Crenewable^{crypto}$	Total capital expenditure on the renewable power installation for using crypto as the virtual energy carrier ($)
$Crenewable^{hyd}$	Total capital expenditure on the renewable power installation for using green hydrogen as a traditional energy carrier ($)
$Ctotal^{crypto}$	Total capital expenditure for using crypto as the virtual energy carrier ($)
$Ctotal^{hyd}$	Total capital expenditure for using green hydrogen as a traditional energy carrier ($)

D(*t*)	Bitcoin network difficulty in different time intervals, represented by the index *t*
Emavoided	Total avoided emissions due to the utilization of renewable power in the conventional mitigation framework (kg CO_2-eq)
$Emavoided^{crypto}$	Total avoided emissions due to the utilization of crypto as the virtual energy carrier (kg CO_2-eq)
$Emavoided^{hyd}$	Total avoided emissions due to the utilization of green hydrogen as a traditional energy carrier (kg CO_2-eq)
Emoriginalcrypto	Total life cycle emissions based on the grid power supply to the crypto mining operations (kg CO_2-eq)
Emoriginalhydrogen	Total life cycle emissions based on different hydrogen pathways in meeting the global hydrogen demand (kg CO_2-eq)
Emtotal	Total life cycle emissions based on utilizing renewable power in the conventional mitigation framework (kg CO_2-eq)
$Emtotal^{crypto}$	Total life cycle emissions based on utilizing the renewable power for crypto as the virtual energy carrier (kg CO_2-eq)
$Emtotal^{hyd}$	Total life cycle emissions based on using green hydrogen as a traditional energy carrier (kg CO_2-eq)
$Emtotal^{neg}$	Total life cycle emissions based on grid electricity import in the negative mitigation framework (kg CO_2-eq)
etotal	Total energy supply based on green hydrogen as a traditional energy carrier (kWh)
h2(*t*)	Total hydrogen production in different time intervals for the conventional mitigation framework, represented by the index *t* (kg)
$h2(t)^{hyd}$	Total hydrogen production in different time intervals for using green hydrogen as a traditional energy carrier, represented by the index *t* (kg)
Ndac	Number of units of DAC units utilized in the negative mitigation framework
Nele	Number of electrolyzer units utilized for the conventional mitigation framework
$Nele^{hyd}$	Number of electrolyzer units utilized for using green hydrogen as a traditional energy carrier
Nhpump	Number of heat pumps utilized for the conventional mitigation framework
$Nhpump^{crypto}$	Number of heat pumps utilized for using crypto as the virtual energy carrier
Nminer	Number of miners utilized for the conventional mitigation framework
$Nminer^{crypto}$	Number of miners utilized for using crypto as the virtual energy carrier
NPV	Net present value from using the multi-pronged approach in the conventional mitigation framework ($)
NPV^{crypto}	Net present value from using crypto as the virtual energy carrier ($)
NPV^{neg}	Net present value from carbon-neutral bitcoins in the negative mitigation framework ($)
Nrenewable	Total capacity for the renewable power installation in the conventional mitigation framework
$Nrenewable^{crypto}$	Total capacity for the renewable power installation for using crypto as the virtual energy carrier
$Nrenewable^{hyd}$	Total capacity for the renewable power installation for using green hydrogen as a traditional energy carrier
$Opexccs^{neg}$	Total operating expenses due to storage and transport for the captured CO_2 in the negative mitigation framework ($)
$Opex^{crypto}$	Total operating cost for using crypto as the virtual energy carrier ($)
Opexdac	Total operating expenses on DAC units in the negative mitigation framework ($)

Opexele	Total operating cost for the green hydrogen infrastructure in the conventional mitigation framework ($)
$Opexele^{hyd}$	Total operating cost due to electrolyzer units for using green hydrogen as a traditional energy carrier ($)
$Opexgrid^{neg}$	Total operating expenses due to power input from respective state electricity grids in the negative mitigation framework ($)
Opexhp(*t*)	Operating cost for the auxiliary heat pumps in different time intervals for the conventional mitigation framework, represented by the index *t* ($)
$Opexhp(t)^{crypto}$	Operating cost for the auxiliary heat pumps in different time intervals for using crypto as the virtual energy carrier, represented by the index *t* ($)
Opexhpump	Total operating cost for the auxiliary heat pumps in the conventional mitigation framework ($)
$Opexhpump^{crypto}$	Total operating cost for the auxiliary heat pumps for using crypto as the virtual energy carrier ($)
$Opex^{hyd}$	Total operating cost for using green hydrogen as a traditional energy carrier ($)
$Opexhyd^{neg}$	Total operating expenses due to green hydrogen power input in the negative mitigation framework ($)
Opexrenew(*t*)	Operating cost for the renewable power installation in different time intervals for the conventional mitigation framework, represented by the index *t* ($)
$Opexrenew(t)^{crypto}$	Operating cost for the renewable power installation in different time intervals for using crypto as the virtual energy carrier, represented by the index *t* ($)
$Opexrenew(t)^{hyd}$	Operating cost for the renewable power installation in different time intervals for using green hydrogen as a traditional energy carrier, represented by the index *t* ($)
Opexrenewable	Total operating cost for the renewable power installation in the conventional mitigation framework ($)
$Opexrenewable^{crypto}$	Total operating cost for the renewable power installation for using crypto as the virtual energy carrier ($)
$Opexrenewable^{hyd}$	Total operating cost for the renewable power installation for using green hydrogen as a traditional energy carrier ($)
$Opextransport^{hyd}$	Total operating cost due to transportation of green hydrogen as a traditional energy carrier ($)
Pavailable(*t*)	Total available power in different time intervals in the conventional mitigation framework, represented by the index *t* (kWh)
$Pavailable(t)^{crypto}$	Total available power in different time intervals for using crypto as the virtual energy carrier, represented by the index *t* (kWh)
$Pavailable(t)^{hyd}$	Total available power in different time intervals for using green hydrogen as a traditional energy carrier, represented by the index *t* (kWh)
$Pdac(t)^{neg}$	Total power utilized in the DAC units for the negative mitigation framework, represented by the index *t* (kWh)
Pele(*t*)	Total power utilized in the green hydrogen infrastructure, represented by the index *t* (kWh)
$Pele(t)^{hyd}$	Total utilized power in different time intervals for using green hydrogen as a traditional energy carrier, represented by the index *t* (kWh)
$Pgrid(t)^{neg}$	Total power input from respective state electricity grids in the negative mitigation framework, represented by the index *t* (kWh)
Pheat(*t*)	Total heat to be removed from the mining equipment, represented by the index *t* (kWh)

Phpump(*t*)	Total power utilized in the auxiliary heat pumps, represented by the index *t* (kWh)
$Phpump(t)^{crypto}$	Total power utilized in the auxiliary heat pumps for using crypto as the virtual energy carrier, represented by the index *t* (kWh)
$Phpump(t)^{neg}$	Total power utilized in the auxiliary heat pumps for the negative mitigation framework, represented by the index *t* (kWh)
$Phydrogen(t)^{neg}$	Total green hydrogen power input in the negative mitigation framework, represented by the index *t* (kWh)
Pminer(*t*)	Total power utilized in the mining equipment, represented by the index *t* (kWh)
$Pminer(t)^{crypto}$	Total power utilized in the mining equipment for using crypto as the virtual energy carrier, represented by the index *t* (kWh)
$Pminer(t)^{neg}$	Total power utilized in the mining equipment for the negative mitigation framework, represented by the index *t* (kWh)
$pnew^{crypto}$	Total renewable power generation which can be funded through using crypto as the virtual energy carrier based on the respective levelized costs (kWh)
Psolar(*t*)	Power output from the solar PV system in different time intervals in the conventional mitigation framework, represented by the index *t* (kWh)
Psurplus(*t*)	Total surplus power in different time intervals in the conventional mitigation framework, represented by the index *t* (kWh)
$Psurplus(t)^{crypto}$	Total surplus power in different time intervals for using crypto as the virtual energy carrier, represented by the index *t* (kWh)
$Psurplus(t)^{hyd}$	Total surplus power in different time intervals for using green hydrogen as a traditional energy carrier, represented by the index *t* (kWh)
$Ptotal(t)^{neg}$	Total power input in the negative mitigation framework, represented by the index *t* (kWh)
Putilized(*t*)	Total utilized power in different time intervals in the conventional mitigation framework, represented by the index *t* (kWh)
$Putilized(t)^{crypto}$	Total utilized power in different time intervals for using crypto as the virtual energy carrier, represented by the index *t* (kWh)
Pwind(*t*)	Power output from the wind turbine in different time intervals in the conventional mitigation framework, represented by the index *t* (kWh)
Revcrypto(*t*)	Revenue component based on crypto operations in different time intervals for the conventional mitigation framework, represented by the index *t* ($)
REVENUE	Total revenue for the project life considered in the conventional mitigation framework ($)
Revhydrogen(*t*)	Revenue component based on green hydrogen supply in different time intervals for the conventional mitigation framework, represented by the index *t* ($)
rsolar(*t*)	Solar irradiation intensity in different time intervals, represented by the index *t* (W/m^2)
SALdac	Salvage value for the DAC units in the negative mitigation framework ($)
SALele	Salvage value for the green hydrogen infrastructure in the conventional mitigation framework ($)
$SALele^{hyd}$	Salvage value for the green hydrogen infrastructure for using green hydrogen as a traditional energy carrier ($)
SALhpump	Salvage value for the auxiliary heat pumps in the conventional mitigation framework ($)
$SALhpump^{crypto}$	Salvage value for the auxiliary heat pumps for using crypto as the virtual energy carrier ($)

SALminer	Salvage value for the mining equipment in the conventional mitigation framework ($)
$SALminer^{crypto}$	Salvage value for the mining equipment for using crypto as the virtual energy carrier ($)
SALrenewable	Salvage value for the renewable power installation for the conventional mitigation framework ($)
$SALrenewable^{crypto}$	Salvage value for the renewable power installation for using crypto as the virtual energy carrier ($)
$SALrenewable^{crypto}$	Salvage value for the renewable power installation for using green hydrogen as a traditional energy carrier ($)
$SALtotal^{crypto}$	Total salvage value for using crypto as the virtual energy carrier ($)
$SALtotal^{crypto}$	Total salvage value for using green hydrogen as a traditional energy carrier ($)
UTL	Utilization percentage from the solar or wind power installation in the conventional mitigation framework (%)
UTL^{crypto}	Utilization percentage from the solar or wind power installation for using crypto as the virtual energy carrier (%)
UTL^{hyd}	Utilization percentage from the solar or wind power installation for using green hydrogen as a traditional energy carrier (%)
x (*j*)	Percentage of miners in a given geographical location, represented by the index *j*
y (*m*)	Percentage of different hydrogen pathways in meeting the global hydrogen demand, represented by the index *m*

S6. Parameters

Table S2. Parameters used in the optimization modeling frameworks for different technological solutions proposed in the study.

Parameters	Value	Unit	Ref.
ACdac	4,069,000	$/MW	(2)
ACele	630,000	$/MW	(3)
AChpump	300,000	$/unit	(4)
ACminer	3,395	$/unit	(5)
ACsolar	1,318.29	$/kW	(6)
ACwind	1,277.45	$/kW	(6)
cfdac	0.04		(7)
cfele	0.03		(8)
cfsolar	0.058	$kgCO_2$-eq/kWh	(9)
cfwind	0.01310	$kgCO_2$-eq/kWh	(9)
COP	3		(10)
cst	10.75	$/$tCO_2$	(11)
ctr	5.17	$/$tCO_2$	(11)
dacmax	1,000	kWh	(2)
dacmin	0	kWh	(2)
effc	0.58		(12)
ele	0.68		(13)
hf	0.38		(7)
hst	3.4	$/kg H_2	(14)
lcap	300	$/$tCO_2$	(15)
LHV	33.33	kWh/kg H_2	(16)
lsolar	36.76	$/MWh	(6)
lwind	27.85	$/MWh	(6)
minerhash	100	TH/s	(5)
opfachp	0.00207	$/kWh	(17)
opfacsolar	22.64	$/kW-yr	(6)

Parameters	Value	Unit	Ref.
opfacwind	43	$/kW-yr	(6)
pemax	1,000	kWh	(7)
pemin	0	kWh	(7)
phmax	1,000	kWh	(7)
phmin	0	kWh	(7)
Pminermax	3.25	kWh	(5)
Pminermin	0	kWh	(5)
R	6.25		(18)
sdac	0.233		(19)
sele	0.05		(19)
shpump	0.05		(19)
sminer	0		(19)
S^{PV}	14,164	m^2	(20)
ssolar	0.471		(19)
swind	0.471		(19)
v_{cin}	2	m/s	(1)
v_{cout}	25	m/s	(1)
v_r	11	m/s	(1)

S7. Sensitivity Analysis

The effectiveness of the proposed technological solutions across various parts of the study hinges on the bitcoin network dynamics. This includes elements such as the average network difficulty and the prevailing selling price. To accommodate these uncertainties, we leverage parametric programming in our investigation to discern the influence of these factors on the efficiency of the proposed solutions. Utilizing archived network parameters, we obtain the spectrum of values that serve as the foundation for our parametric sensitivity assessment. Based on the utilization of studied frameworks, we can potentially avoid carbon emissions associated with bitcoin mining and hydrogen production. Thus, in addition to the network dynamics, we have included the impact of carbon credits. The parametric sensitivity analysis begins by identifying the states with the highest efficiency for a given technological solution under the base case evaluation. For instance, it was observed that under the multi-pronged strategy, the solar power facility in New Mexico and the wind energy system in Wyoming had the highest economic potential to fund the new renewable capacity at the considered base case values for selling price, network difficulty, and the issued carbon credits. Accordingly, these two states were chosen for the parametric study calculations, as depicted in Figure 3. Subsequently, we analyze how the varying bitcoin prices and the issued carbon credits for the avoided emissions would impact the effectiveness of the proposed multi-pronged strategy in New Mexico and Wyoming. The same analysis with regard to the prices and issued carbon credits has also been performed at a lower network difficulty, which would correspond to easier mining conditions. However, we also conduct a detailed analysis of the impact of network difficulty and examine easier and computationally expensive mining conditions (corresponding to higher recorded network difficulties) in conjunction with the bitcoin prices. Similarly, for the negative mitigation framework, it was observed that Idaho had the highest economic potential under the base case evaluation without any incentives for the green hydrogen power supply. Accordingly, we conduct the sensitivity analysis, illustrated in Figure 6, to study how the varying carbon credits, bitcoin prices, and the degree of incentivization for the green hydrogen power supply would impact the economic potential of bitcoin mined to fund the capture of CO_2. Lastly, it was crucial to study how the potential of bitcoin mining is impacted in states with high contributions of fossil resources in their state electricity grid. Hence, we identify West Virginia has a significant contribution of fossil resources in its grid power supply. Thus, we conduct the parametric sensitivity analysis on the varying selling prices and the degree of incentivization in the green hydrogen power supply.

Table S3 depicts the equipment specifications utilized in the evaluation of the proposed technological solutions. In this work, we conduct sensitivity analysis to investigate the impact of equipment specifications on the efficiency of the proposed technological solutions. Figures S1 and S2 depict the sensitivity analysis results for solar and wind power increment potential in the conventional mitigation framework across different US states. We begin the analysis by illustrating the influence of mining equipment hash rates on the conventional mitigation framework. Figure S1a shows that under the minimum hash rate for the bitcoin mining equipment, states such as New Mexico maintain a capacity increment potential close to the base case evaluation, indicating considerable resilience to less efficient mining operations. Similarly, the wind capacity increment potential decreases by 19% in Wyoming based on the reduction in mining equipment hash rate, as shown in Figure S2a. On the other hand, states like Arizona, California, Hawaii, etc., can utilize solar energy resources to enhance the increment potential by more than 100% in case of maximum mining equipment hash rates. Correspondingly, Ohio, Pennsylvania, Washington, etc., can capitalize on the increase in mining equipment hash rate to significantly enhance wind capacity increment potential. In the case of electrolyzer efficiency, the solar capacity increment potential was reduced by 3.4% and 11.9% in Idaho and South Dakota, respectively. However, many states indicated a relatively smaller effect on the increment potential due to electrolyzer efficiency. Therefore, the impact on the solar capacity increment potential due to electrolyzer efficiency is less significant than the mining equipment hash rate, which can be attributed to the load balance between green hydrogen production and bitcoin mining. However, the maximum impact on the wind capacity potential due to electrolyzer efficiency was observed in Michigan, indicating a higher reliance on green hydrogen production. Figures S1e and f depict the variability in solar capacity potential with respect to heat pump performance. It was observed that the change in the solar power increment potential varies between -55% to 197.5% based on the heat pump performance. Similarly, Figures S2e and f demonstrate the effect of heat pump performance on wind capacity potential. In the case of maximum heat pump performance, the results indicate that states such as Nebraska and Colorado can considerably increase the wind capacity increment potential in the proposed conventional mitigation framework. The sensitivity analysis results in the conventional mitigation framework highlight the dependencies between specific equipment parameters and the solar and wind power increment potential across US states. The findings suggest the need for strategic investment in mining equipment, electrolyzers, and heat pumps to leverage the solar and wind capacity increment potential, particularly in states that could benefit most from such technological enhancements.

Table S3. Equipment specifications utilized in the evaluation of the proposed technological solutions.

Equipment	Specification	Value	Unit	Ref.
Bitcoin Miner	Hashrate	100	TH/s	(5)
Electrolyzer	Efficiency	68	%	(13)
Heat Pump	COP	3	-	(10)
DAC	Electrical/Heating demand	0.349/1.977	kWh/kgCO_2	(21)

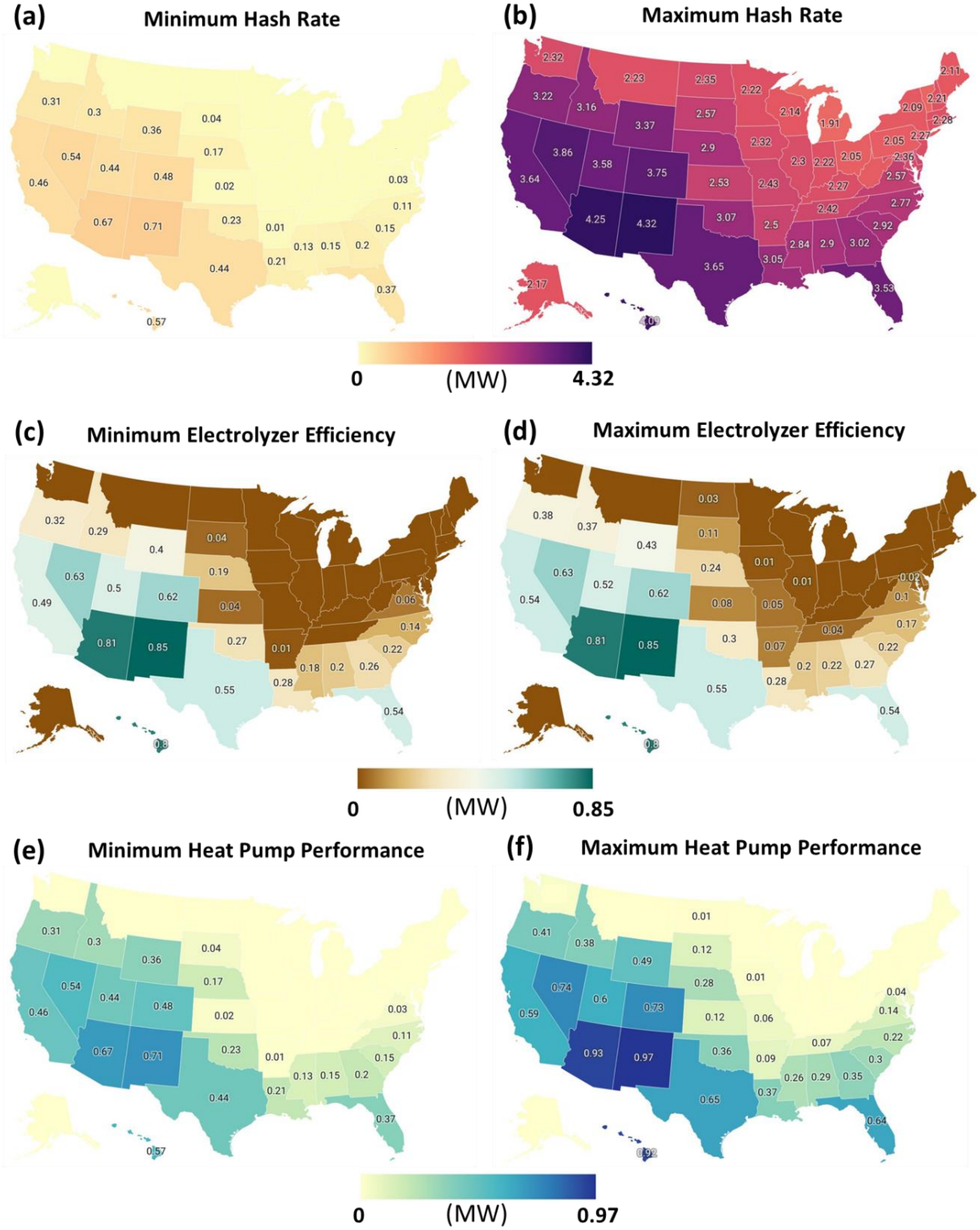

Figure S1. Sensitivity analysis results for solar power increment potential in the conventional mitigation framework based on initial solar power capacity. a., Total increment potential for solar capacity (MW) for the conventional mitigation framework in different US states based on minimum mining equipment hash rate. b., Total increment potential for solar capacity (MW) for the conventional mitigation framework in different US states based on maximum mining equipment hash rate. c., Total increment potential for solar capacity (MW) for the conventional mitigation framework in different US states based on minimum electrolyzer efficiency. d., Total increment potential for solar capacity (MW) for the conventional mitigation framework in different US states

based on maximum electrolyzer efficiency. e., Total increment potential for solar capacity (MW) for the conventional mitigation framework in different US states based on minimum heat pump performance. f., Total increment potential for solar capacity (MW) for the conventional mitigation framework in different US states based on maximum heat pump performance.

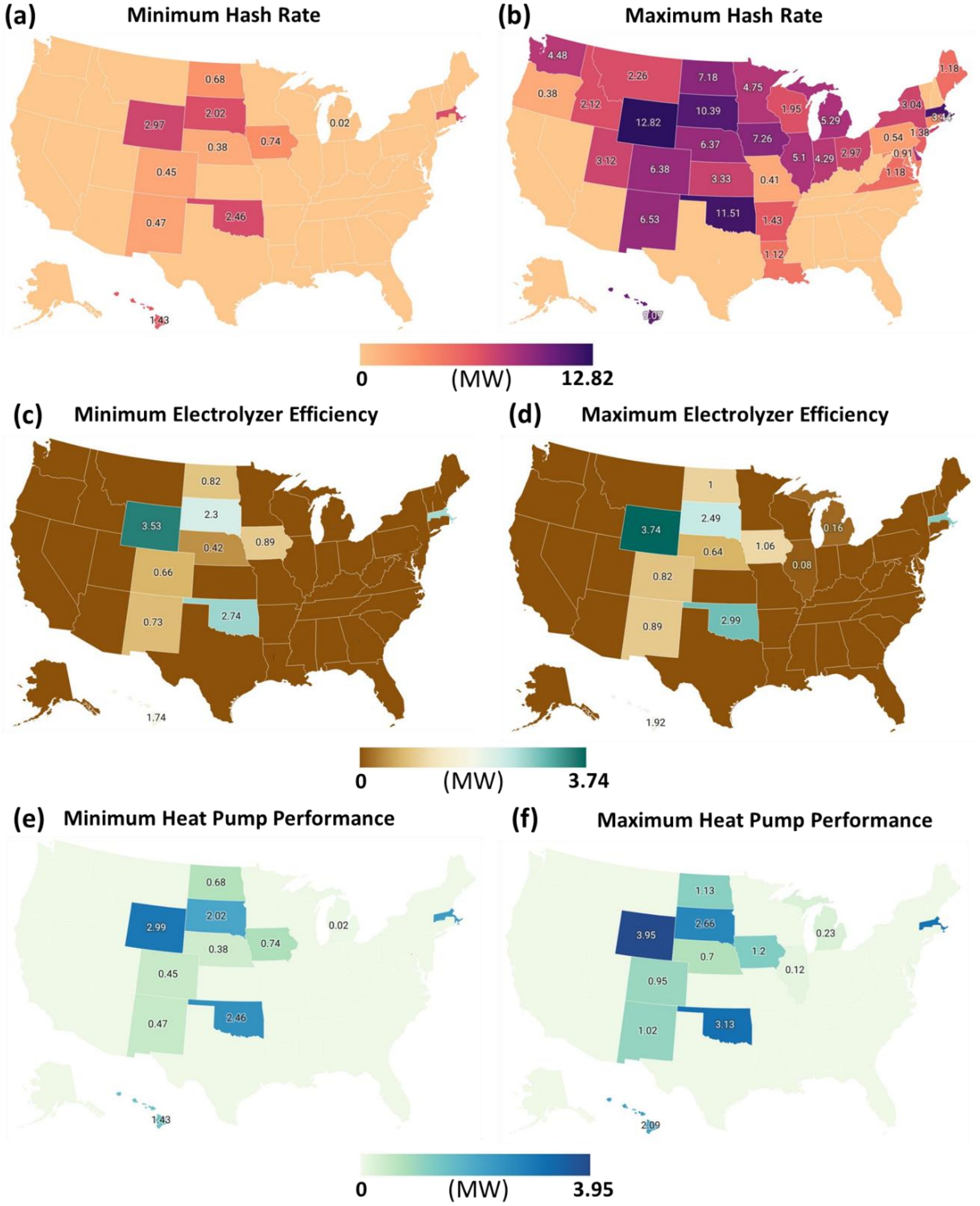

Figure S2. Sensitivity analysis results for wind power increment potential in the conventional mitigation framework based on initial wind power capacity. a., Total increment potential for wind power capacity (MW) for the conventional mitigation framework in different US states based on minimum mining equipment hash rate. b., Total increment potential for wind power capacity (MW)

for the conventional mitigation framework in different US states based on maximum mining equipment hash rate. c., Total increment potential for wind power capacity (MW) for the conventional mitigation framework in different US states based on minimum electrolyzer efficiency. d., Total increment potential for wind power capacity (MW) for the conventional mitigation framework in different US states based on maximum electrolyzer efficiency. e., Total increment potential for wind power capacity (MW) for the conventional mitigation framework in different US states based on minimum heat pump performance. f., Total increment potential for wind power capacity (MW) for the conventional mitigation framework in different US states based on maximum heat pump performance.

The sensitivity analysis results for the virtual carrier framework highlight the variation in solar and wind energy potential for each bitcoin based on changing specifications for mining equipment hash rates and heat pump performance, as depicted in Figures S3 and S4. It was observed that the percentage change in solar energy potential for each bitcoin significantly varies based on variations in mining equipment hash rate. Decreasing the mining equipment hash rate negatively affects the solar and wind energy potential in the virtual carrier framework. States such as New Mexico and Wyoming, which had illustrated resilience against decreasing mining equipment hash rate in the conventional mitigation framework, cannot attain renewable energy potential in the virtual carrier framework. However, it is important to note that states such as Massachusetts can also attain a 117% improvement in wind energy potential based on the increase in mining equipment hash rate, highlighting the opportunity to leverage technological improvements for efficiency gains in the virtual carrier framework. The heat pump performance also shows a considerable impact on the virtual carrier framework. The average increment in solar and wind energy potential in the virtual carrier framework based on increasing heat pump performance is 16.8% and 6.3%, respectively. Higher heat pump performance allows more power utilization toward bitcoin mining operations, enhancing the economic potential generated to be utilized for renewable energy output. The findings from the sensitivity analysis highlight the opportunity for targeted investments in technological advancements to enhance the solar and wind energy potential using the virtual carrier framework. By improving the performance metrics of mining equipment and heat pump technologies, different states can enhance solar and wind energy production for each bitcoin mined and contribute toward renewable energy targets.

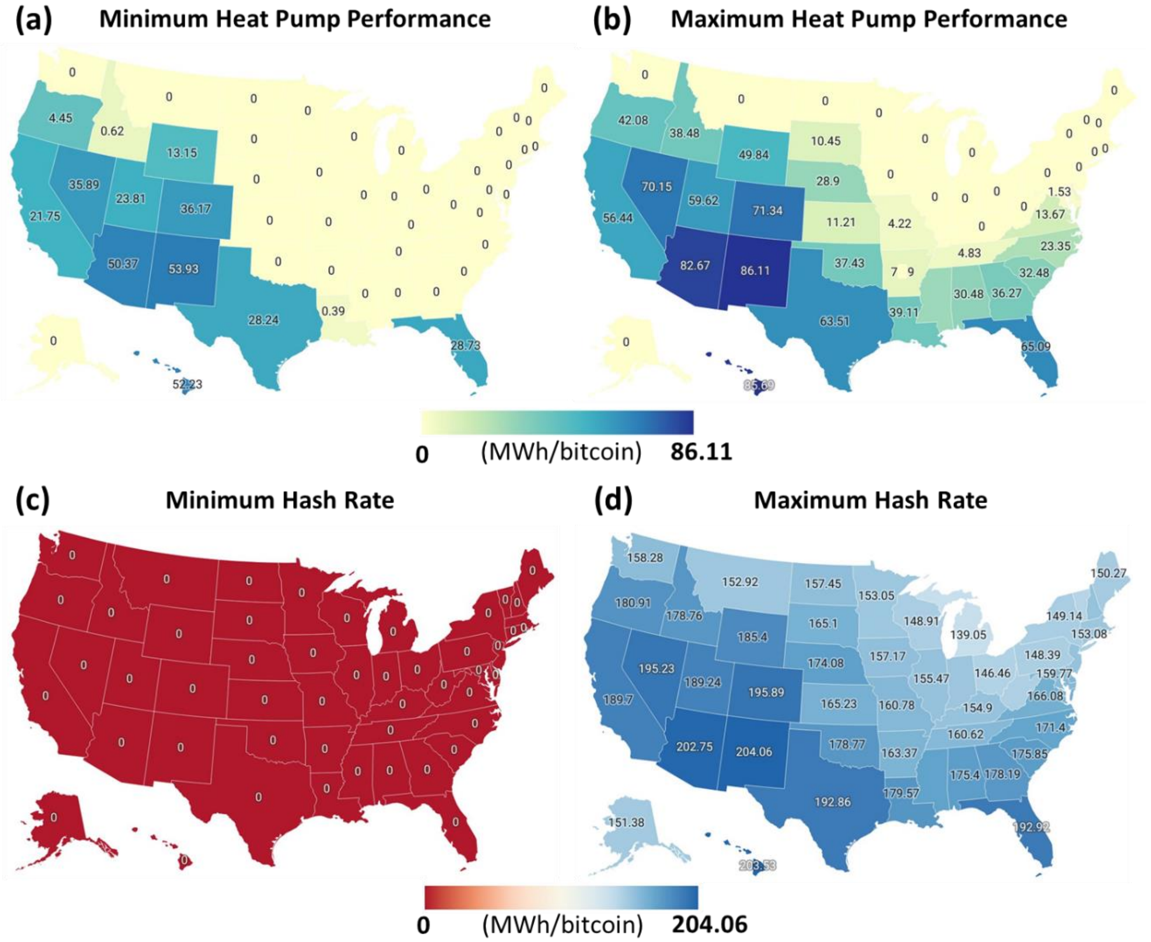

Figure S3. Sensitivity analysis results for the virtual carrier framework based on solar power generation potential. a., Solar power generation potential for each bitcoin mined (MWh/bitcoin) for the virtual carrier framework in different US states based on minimum mining equipment hash rate. b., Solar power generation potential for each bitcoin mined (MWh/bitcoin) for the virtual carrier framework in different US states based on maximum mining equipment hash rate. c., Solar power generation potential for each bitcoin mined (MWh/bitcoin) for the virtual carrier framework in different US states based on minimum heat pump performance. d., Solar power generation potential for each bitcoin mined (MWh/bitcoin) for the virtual carrier framework in different US states based on maximum heat pump performance.

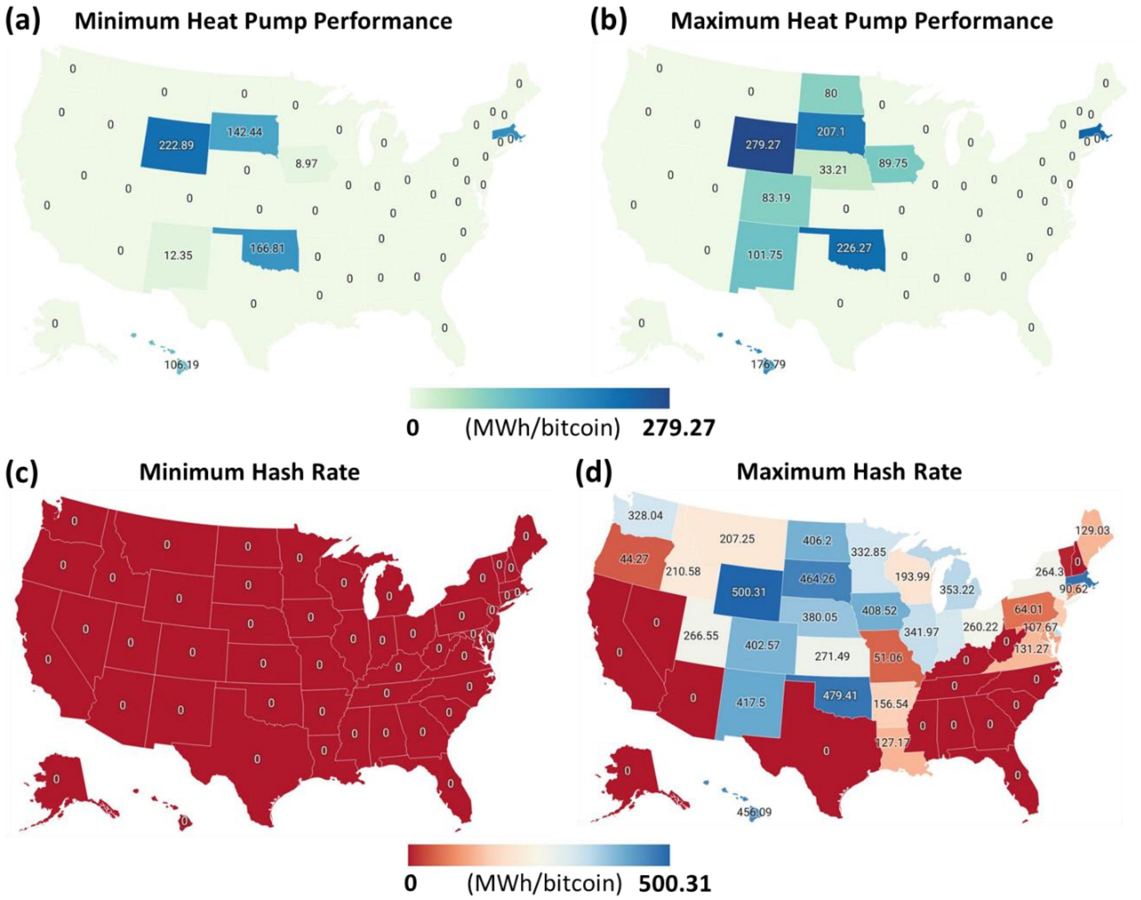

Figure S4. Sensitivity analysis results for the virtual carrier framework based on wind power generation potential. a., Wind power generation potential for each bitcoin mined (MWh/bitcoin) for the virtual carrier framework in different US states based on minimum mining equipment hash rate. b., Wind power generation potential for each bitcoin mined (MWh/bitcoin) for the virtual carrier framework in different US states based on maximum mining equipment hash rate. c., Wind power generation potential for each bitcoin mined (MWh/bitcoin) for the virtual carrier framework in different US states based on minimum heat pump performance. d., Wind power generation potential for each bitcoin mined (MWh/bitcoin) for the virtual carrier framework in different US states based on maximum heat pump performance.

Figure S5 depicts the sensitivity analysis results for the negative mitigation framework based on equipment specifications. Similar to conventional mitigation framework and virtual carrier operations, higher mining equipment hash rates correspond to increased negative mitigation potential for each bitcoin mined. For instance, in the case of Idaho, which emerged as the most favorable alternative, a 107% increase in negative mitigation potential can be attained using hash rate improvement in the mining equipment. The DAC energy consumption also emerges as a pivotal factor that influences the effectiveness of the negative mitigation framework. Considering the load balance requirements, lower specific energy consumption in DAC corresponds to higher energy utilization in the mining equipment, increasing the economic potential, which can be used to enhance the carbon capture capacity. States such as North Dakota, which were not feasible in the negative mitigation framework, can achieve significant improvement in the negative mitigation potential based on decreasing the specific energy requirements in DAC. However, even with the improved performance of DAC, the negative mitigation framework was not feasible in some states such as West Virginia, Indiana, etc., which can be attributed to the high fossil dependence of the grid power supply. Moreover, the performance of heat pumps also varies the effectiveness of the

negative mitigation framework. Based on improvements in heat pump performance, some states, such as Arizona and Nebraska, can enhance the negative mitigation potential by more than 100%. However, it is equally important to note that due to a decrease in heat pump performance, the negative mitigation framework becomes infeasible in some states such as Florida, Georgia, Illinois, etc. The findings from the sensitivity analysis of the negative mitigation framework depict the importance of a tailored approach to gain maximum benefits in carbon capture capacity enabled using crypto operations. The energy profile corresponding to each state aligned with technological advancement to improve the equipment performance metrics can enhance the economic potential utilized to increase the carbon capture capacity.

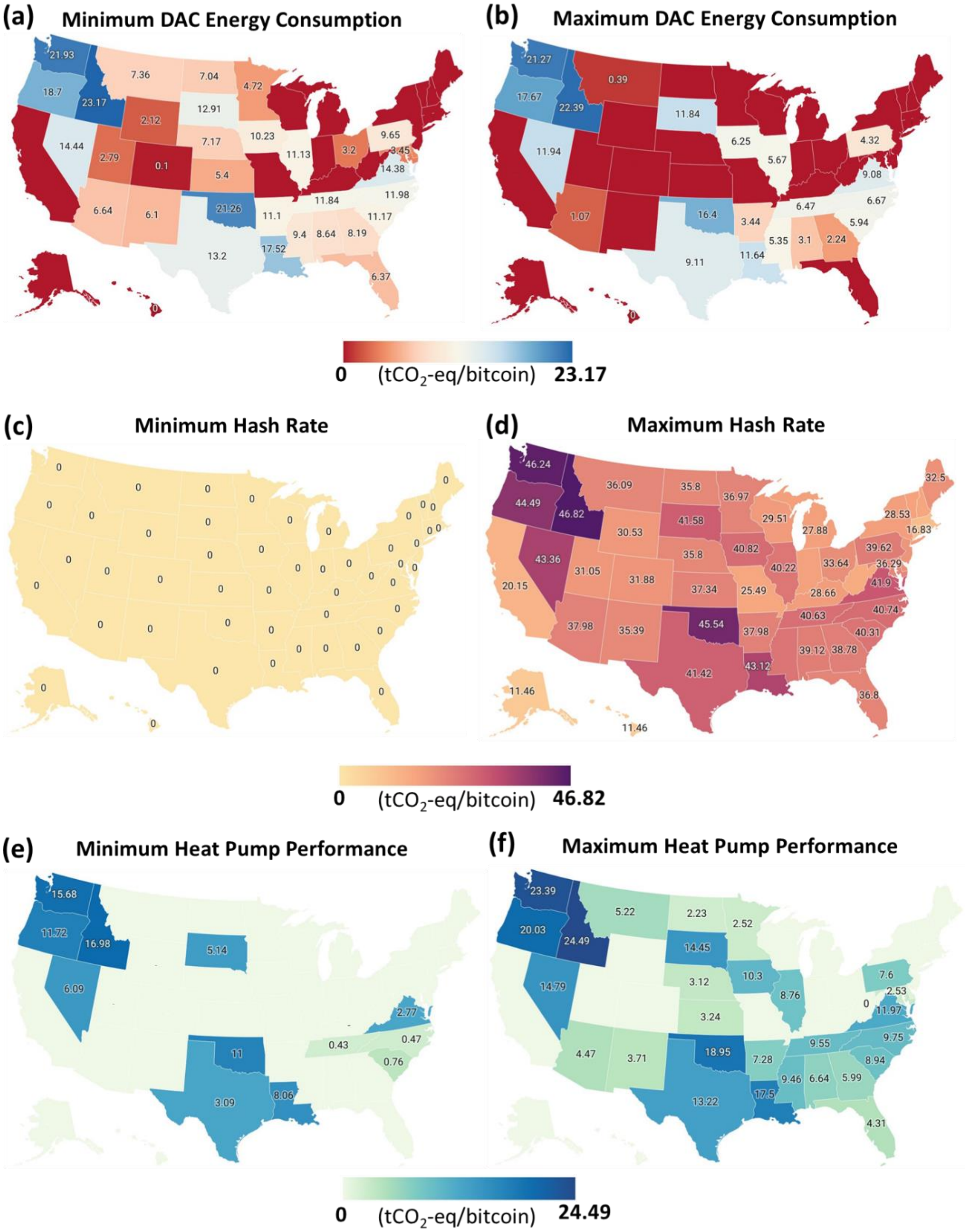

Figure S5. Sensitivity analysis results for negative mitigation potential. a., Negative mitigation potential for each bitcoin mined (tCO_2-eq/bitcoin) in different US states based on minimum mining equipment hash rate. b., Negative mitigation potential for each bitcoin mined (tCO_2-eq/bitcoin) in different US states based on maximum mining equipment hash rate. c., Negative mitigation potential for each bitcoin mined (tCO_2-eq/bitcoin) in different US states based on minimum electrolyzer efficiency. d., Negative mitigation potential for each bitcoin mined (tCO_2-eq/bitcoin) in different US states based on maximum electrolyzer efficiency. e., Negative mitigation potential for each bitcoin mined (tCO_2-eq/bitcoin) in different US states based on minimum heat pump

performance. f., Negative mitigation potential for each bitcoin mined (tCO_2-eq/bitcoin) in different US states based on maximum heat pump performance.

Figure S6 illustrates the Pareto-optimal curves for the proposed technological solutions depicting the NPV and GWP values. The points with the maximum NPV correspond to the highest solar and wind capacity increment potential in the conventional mitigation framework, as shown in Figures S6a and b. Similarly, Figures S6c and d highlight the optimal solutions for maximum renewable energy potential based on the utilization of crypto operations as virtual energy carriers.

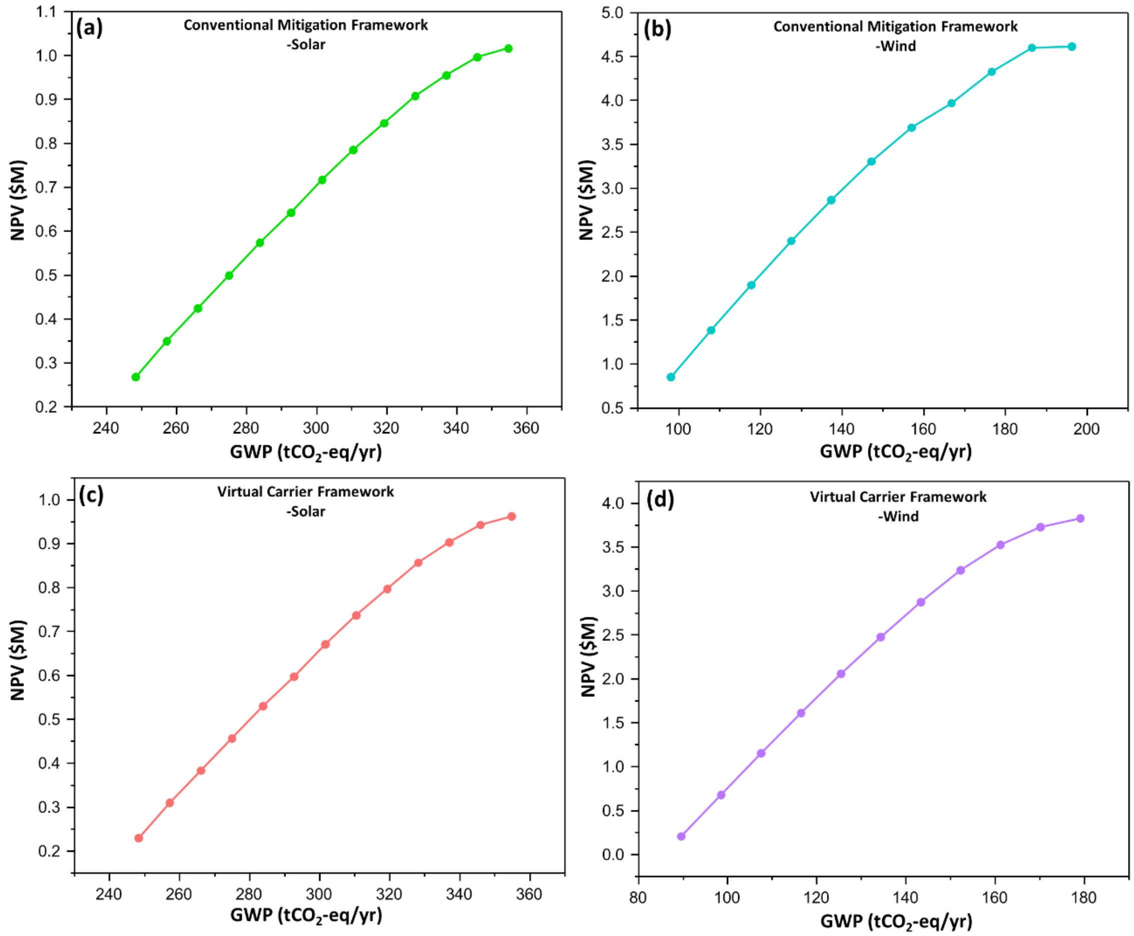

Figure S6. Pareto-optimal curves depicting the economic and environmental performances for proposed technological solutions: a., Conventional mitigation framework for solar power capacity increment. b., Conventional mitigation framework for wind power capacity increment. c., Virtual carrier framework for solar power generation potential. d., Virtual carrier framework for wind power generation potential.

SI References

1. M. H. Shams *et al.*, Optimal operation of electrical and thermal resources in microgrids with energy hubs considering uncertainties. *Energy* **187**, 115949 (2019).
2. D. Slesinski, S. Litzelman, How Low-Carbon Heat Requirements for Direct Air Capture of CO_2 Can Enable the Expansion of Firm Low-Carbon Electricity Generation Resources. *Frontiers in Climate* **3**, 728719 (2021).
3. M. Fasihi, R. Weiss, J. Savolainen, C. Breyer, Global potential of green ammonia based on hybrid PV-wind power plants. *Applied Energy* **294**, 116170 (2021).

4. J. Cox, S. Belding, T. Lowder, Application of a novel heat pump model for estimating economic viability and barriers of heat pumps in dairy applications in the United States. *Applied Energy* **310**, 118499 (2022).
5. S19 Specifications, Bitmain (2022). https://support.bitmain.com/hc/en-us/articles/900000253583-S19-Specifications. Accessed 26 December 2023.
6. L. Vimmerstedt *et al.*, Annual Technology Baseline: The 2022 Electricity Update. National Renewable Energy Laboratory, (2022).
7. H. Niaz, M. H. Shams, J. Liu, F. You, Mining Bitcoins with Carbon Capture and Renewable Energy for Carbon Neutrality Across States in the USA. *Energy & Environmental Science* **15**, 4426-4426 (2022).
8. D. Jang, J. Kim, D. Kim, W.-B. Han, S. Kang, Techno-economic analysis and Monte Carlo simulation of green hydrogen production technology through various water electrolysis technologies. *Energy Conversion and Management* **258**, 115499 (2022).
9. D. FitzGerald, T. Sonderegger, Documentation of changes implemented in the ecoinvent database v3. 9.1. (2022).
10. D. Gibb, J. Rosenow, R. Lowes, N. J. Hewitt, Coming in from the cold: Heat pump efficiency at low temperatures. *Joule* **7**, 1939-1942 (2023).
11. E. Smith *et al.*, The cost of CO_2 transport and storage in global integrated assessment modeling. *International Journal of Greenhouse Gas Control* **109**, 103367 (2021).
12. S. Mekhilef, R. Saidur, A. Safari, Comparative study of different fuel cell technologies. *Renewable and Sustainable Energy Reviews* **16**, 981-989 (2012).
13. V. A. Martinez Lopez, H. Ziar, J. W. Haverkort, M. Zeman, O. Isabella, Dynamic operation of water electrolyzers: A review for applications in photovoltaic systems integration. *Renewable and Sustainable Energy Reviews* **182**, 113407 (2023).
14. J. Collis, R. Schomäcker, Determining the Production and Transport Cost for H_2 on a Global Scale. *Frontiers in Energy Research* **10**, 909298 (2022).
15. K. S. Lackner, H. Azarabadi, Buying down the Cost of Direct Air Capture. *Industrial & Engineering Chemistry Research* **60**, 8196-8208 (2021).
16. G. Brändle, M. Schönfisch, S. Schulte, Estimating long-term global supply costs for low-carbon hydrogen. *Applied Energy* **302**, 117481 (2021).
17. E. Popovski, T. Fleiter, H. Santos, V. Leal, E. O. Fernandes, Technical and economic feasibility of sustainable heating and cooling supply options in southern European municipalities-A case study for Matosinhos, Portugal. *Energy* **153**, 311-323 (2018).
18. A. de Vries, Bitcoin boom: What rising prices mean for the network’s energy consumption. *Joule* **5**, 509-513 (2021).
19. Depreciation Calculator, Calculator.net (2022). https://www.calculator.net/depreciation-calculator.html. Accessed 26 December 2023.
20. S. Ong, C. Campbell, P. Denholm, R. Margolis, G. Heath, Land-use requirements for solar power plants in the United States. National Renewable Energy Laboratory, (2013).
21. CCUS in Clean Energy Transitions, International Energy Agency (2020).
